# Single‐Cell Transcriptomic Analysis Reveals an Inflammatory Antigen‐Presenting Macrophages Subtype Drive Vitiligo Pathogenesis Through STAT1‐Mediated Dual Mechanisms

**DOI:** 10.1155/mi/8878698

**Published:** 2025-12-22

**Authors:** Ruozhou Qi, Min Huang, Ziyi Lin, Huanhuan Deng, Rule Sa, Yi Chen, Guangshan Chen, Xingwu Duan

**Affiliations:** ^1^ Department of Dermatology, Dongzhimen Hospital, Beijing University of Chinese Medicine, Beijing, China, bucm.edu.cn

**Keywords:** macrophages, melanocytes, single-cell RNA sequencing, STAT1, vitiligo

## Abstract

**Background:**

Vitiligo is a common depigmentary disorder characterized by progressive melanocyte (MEL) loss. While T‐cell activation is central to its pathogenesis, the role of macrophages remains poorly understood. This study characterizes macrophage heterogeneity and function in vitiligo using single‐cell transcriptomic analysis and experimental validation.

**Methods:**

We analyzed single‐cell RNA sequencing (scRNA‐seq) data from healthy and vitiligo‐affected skin to identify macrophage subpopulations. Computational analyses included cell subpopulation clustering, pseudotime trajectory inference, cell–cell communication, and high‐dimensional weighted gene coexpression network analysis (hdWGCNA). In vivo and in vitro experiments examined the effects of STAT1 suppression on the macrophage inflammatory phenotype and antigen presentation capacity.

**Results:**

scRNA‐seq analysis identified macrophages and T cell subsets enriched in vitiligo. Macrophage subclustering identified five subpopulations, with inflammatory antigen‐presenting macrophages (Mac‐InflamAP) significantly enriched in vitiligo lesions and M1‐polarized. Pseudotime analysis revealed Mac‐InflamAP as a terminal differentiation state. Cell–cell communication analysis showed Mac‐InflamAP exerts TNF‐mediated inhibitory effects on MELs while enhancing T‐cell antigen presentation, thereby promoting MEL loss. hdWGCNA identified STAT1 as a key regulator highly expressed in Mac‐InflamAP. In vivo, STAT1 inhibition by fludarabine ameliorated vitiligo progression by suppressing T cell activation and macrophage M1‐polarization. In vitro experiments confirmed STAT1 suppression reduced macrophage M1 polarization, inflammatory phenotype, and antigen presentation capabilities.

**Conclusions:**

This study reveals an uncharacterized inflammatory macrophage subpopulation crucial to vitiligo pathogenesis through dual mechanisms: direct MEL inhibition and enhanced T‐cell activation. The identification of STAT1 as a key regulatory molecule provides a novel therapeutic target for vitiligo. These findings advance our understanding of immune‐mediated mechanisms in vitiligo.

## 1. Introduction

Vitiligo is a common acquired depigmentary disorder affecting approximately 0.5%–2% of the global population, with profound psychological and social impacts worldwide [1]. The disease is characterized by the progressive loss of melanocytes (MELs), resulting in well‐defined depigmented patches on the skin and mucous membranes [2, 3]. Current knowledge of vitiligo pathogenesis highlights a multifactorial process involving genetic predisposition, environmental triggers, and immune dysregulation, which together drive MEL destruction through both innate and adaptive immune mechanisms [4–6]. Extensive evidence has established that autoreactive cytotoxic T cells are central mediators of MEL damage [7]. Moreover, dysregulated signaling pathways, such as type I interferon (IFN‐*γ*) signaling that facilitates CD8^+^ T‐cell recruitment and activation [8, 9], as well as aberrant antigen expression in MELs [10], have been implicated in disease progression.

Despite these advances, focusing solely on single‐cell types or isolated molecular pathways provides only a fragmented understanding of vitiligo. The disease arises from dynamic and multilayered interactions among immune and epidermal cells, suggesting that analyses of complex molecular networks may yield deeper insights into its pathogenesis and therapeutic opportunities. Among immune cells, macrophages warrant particular attention. Beyond their established role as antigen‐presenting cells that regulate T‐cell responses, macrophages are also indispensable for maintaining tissue integrity, remodeling the skin microenvironment, and sustaining inflammatory cascades [11, 12]. However, their contribution to vitiligo pathogenesis remains largely underexplored, representing a critical gap in current research.

Macrophages constitute a highly heterogeneous population with remarkable functional plasticity. Traditionally, they have been divided into classically activated (M1) and alternatively activated (M2) phenotypes, with M1 macrophages linked to proinflammatory responses and tissue damage and M2 macrophages associated with tissue repair and immunoregulation [13]. Nevertheless, this binary paradigm is overly simplistic, as macrophage activation occurs along a continuum of intermediate and context‐specific states. The advent of single‐cell RNA sequencing (scRNA‐seq) has revolutionized the study of macrophage biology by providing unprecedented resolution of cellular heterogeneity. Recent scRNA‐seq studies have identified disease‐specific macrophage subsets in diverse pathological contexts, including tumor‐associated macrophages in cancer, resident macrophages in neurodegeneration, and inflammatory macrophages in autoimmune disorders [14–16]. Advanced computational approaches, such as subpopulation clustering, pseudotime trajectory inference, cell–cell communication analysis, and high‐dimensional weighted gene coexpression network analysis (hdWGCNA), have further delineated the molecular programs governing macrophage function and intercellular interactions.

Building on these methodological advances, the present study comprehensively characterizes macrophage heterogeneity in vitiligo through integrated scRNA‐seq analysis. By analyzing scRNA‐seq data from healthy and vitiligo‐affected skin, we identified a disease‐enriched inflammatory antigen‐presenting macrophage (Mac‐InflamAP) subset with distinct molecular and functional features. This subpopulation displayed enhanced antigen presentation capacity, promoted T‐cell activation, and exerted TNF‐mediated inhibitory effects on MELs. HdWGCNA further highlighted four regulatory genes—*STAT1*, *VAMP5*, *LAP3*, and *TYMP*—selectively upregulated in Mac‐InflamAP cells. Functional validation in a vitiligo mouse model and in vitro assays confirmed that pharmacological inhibition of STAT1 alleviates disease progression by suppressing macrophage‐driven inflammation. Collectively, our findings uncover a pathogenic macrophage program that contributes to MEL destruction in vitiligo and provide a rationale for targeting macrophage‐specific pathways in therapeutic development.

## 2. Materials and Methods

### 2.1. Data Collection

Publicly available scRNA‐seq data from five healthy donor skin samples and 10 vitiligo‐affected skin biopsies were obtained from the Genome Sequence Archive (GSA) under Accession Number PRJCA006797 (https://ngdc.cncb.ac.cn/bioproject/browse/PRJCA006797) [4]. The independent scRNA‐seq dataset (GEO: GSE288871, https://www.ncbi.nlm.nih.gov/geo/query/acc.cgi?acc=GSE288871) was processed using the same pipeline as the discovery cohort.

### 2.2. scRNA‐seq Analysis

scRNA‐seq data were analyzed using Seurat (v5.3.0) in R (v4.5.0) [17]. Cells with fewer than 500 detected genes, total UMI counts <1000, or mitochondrial gene content >20% were excluded. Genes expressed in fewer than three cells were also filtered out. Data were log‐normalized and scaled, and highly variable genes were identified using FindVariableFeatures. Principal component analysis (PCA) was performed on the top 3000 variable genes, and the first 20 PCs were selected for clustering and uniform manifold approximation and projection (UMAP) visualization. Clustering was performed with FindClusters at a resolution of 0.4. Cell types were annotated based on canonical markers: MELs (MLANA, TYR), keratinocytes (KRTs) (KRT14, KRT10), T cells (CD3D and CD3E), macrophages (CD68 and CD163), Langerhans cells (LCs) (CD1Aand Langerin), fibroblasts (FIBs) (COL1A1 and COL3A1), endothelial cells (ECs) (PECAM1 and VWF), and smooth muscle cells (SMCs) (ACTA2 and MYH11). Differential expression was analyzed using the Wilcoxon rank‐sum test, with significance defined as adjusted *p* < 0.05 and |log_2_FC| > 0.25. The independent scRNA‐seq dataset (GEO: GSE288871) was processed using the same pipeline as the discovery cohort. The observed/expected (Ro/e) ratio was used to quantify cell‐type enrichment, with values >1 indicating enrichment and <1 indicating depletion [18].

### 2.3. Macrophage Subpopulation Analysis

Macrophages were extracted and reclustered using the same procedures, with resolution set to 0.15. Subpopulations were characterized by differential expression and functional annotation. Macrophage subclustering in the validation dataset was performed using the same analytical pipeline and canonical marker genes as in the primary cohort. The transcriptional similarity between macrophage subtypes across the two scRNA‐seq datasets was assessed using Pearson’s correlation analysis.

#### 2.3.1. Gene Set Scoring Was Performed With *AddModuleScore*

TNF signaling (HALLMARK_TNFA_SIGNALING_VIA_NFKB) was obtained from MSigDB, while M1/M2 polarization gene sets were adopted from a published dataset [13]. MHC‐I and MHC‐II scores were calculated using HLA Class I (HLA‐A, HLA‐B, and HLA‐C) and Class II (HLA‐DRA, HLA‐DRB1, HLA‐DQA1, and HLA‐DQB1) genes.

#### 2.3.2. Pseudotime Trajectory Analysis Was Performed in Two Steps

First, CytoTRACE2 was used to determine differentiation states [19]. Next, Monocle3 (v1.3.1) was applied to construct trajectories [20]. Cells were embedded in UMAP space, trajectories were learned with learn_graph, and pseudotime was calculated with order_cells, rooted in CytoTRACE2 results. Pseudotime trajectories were then inferred using Monocle3 with the parameter use_partition = TRUE. This setting ensures that the algorithm focuses on the largest connected component of cells, representing the main, continuous differentiation path. Cells that are isolated, belong to very small clusters, or are spatially disconnected were assigned to separate partitions and excluded from the trajectory calculation. These excluded cells are still visualized in the UMAP embedding but are not assigned pseudotime values, preventing them from introducing noise into the trajectory inference. Dynamic gene expression was assessed using graph_test with q < 1e‐18 and Moran’s *I* > 0.1. Temporal patterns were clustered hierarchically, and cluster‐enriched genes were used for functional enrichment. Gene Ontology (GO) enrichment was performed with clusterProfiler (v4.16.0), with adjusted *p* < 0.05 considered significant [21].

### 2.4. Cell–cell Communication Analysis

Cell–cell communication was inferred with CellChat (v2.1.2) [22]. Separate networks were constructed for healthy and vitiligo skin and then compared to identify condition‐specific changes. Communication probabilities were calculated with computeCommunProb. Interaction numbers and strengths were compared with compareInteractions. Information flow for pathways and ligand–receptor pairs was ranked with rankNet. Dysfunctional signaling was visualized using netVisual_bubble.

### 2.5. hdWGCNA

hdWGCNA was used to build gene coexpression networks [23]. A soft‐thresholding power of 8 was applied to achieve scale‐free topology. Modules were visualized with Lipo plots. Hub genes were defined by module membership (kME > 0.7), differential expression (log_2_FC > 0.5), and protein–protein interaction (PPI) degree (>7).

### 2.6. Western Blot

For protein extraction, mouse skin tissues were excised, weighed, and minced into small pieces. Tissues were then homogenized in ice‐cold RIPA lysis buffer containing protease inhibitors using a tissue homogenizer. Cultured cells were washed with PBS and lysed in the same buffer. The lysates were centrifuged at 12,000 × *g* for 20 min at 4°C to remove debris, and the supernatant was collected. Protein concentration was determined using a BCA Protein Assay Kit (Beyotime). Equal protein amounts (30–50 μg) were separated by SDS–PAGE and transferred onto PVDF membranes. Membranes were blocked with 5% nonfat milk and incubated overnight at 4°C with primary antibodies against Stat1 (1:1000, Cat# 14994, Cell Signaling Technology, CST), phospho‐Stat1 (1:1000, Cat# 9167, CST), Bax (1:1000, Cat# ab32503, Abcam), Bcl‐2 (1:1000, Cat# ab196495, Abcam), Caspase‐3 (1:1000, Cat# ab184787, Abcam), cleaved Caspase‐3 (1:1000, Cat# 68773‐1‐Ig, Proteintech), and *β*‐actin (1:3000, Cat# 66009‐1‐Ig, Proteintech). After washing, membranes were incubated with HRP‐conjugated secondary antibodies for 1 h. Bands were visualized by enhanced chemiluminescence (ECL) and quantified using ImageJ. All experiments were repeated three times.

### 2.7. Quantitative Real‐Time PCR (qRT‐PCR)

Total RNA from mouse skin tissues and cultured cells was extracted using TRIzol reagent and reverse transcribed into cDNA. qPCR was performed with SYBR Green Master Mix on a CFX96 Real‐Time PCR System (Jiangsu Province Nowizan Co., Ltd). Primer sequences are listed in Supporting Information 1: Table S1. Relative gene expression was calculated using the 2^−*ΔΔ*Ct^ method with Gapdh as the reference gene. Each sample was run in triplicate.

### 2.8. Histology and Immunostaining

Dorsal skin tissues were collected at Week 8, fixed in 4% paraformaldehyde, embedded in paraffin, and sectioned (5 μm). For hematoxylin–eosin (H&E) staining, sections were deparaffinized, rehydrated, and stained to evaluate epidermal and dermal morphology. For immunohistochemistry, antigen retrieval was performed in citrate buffer (pH 6.0), followed by blocking with 5% BSA. Sections were incubated overnight at 4°C with primary antibodies against CD4 (1:100, Cat #25229, CST), CD8 (1:200, Cat #98941, CST), F4/80 (1:200, Cat #70076, CST), and CD86 (1:300, Cat #19589, CST). After washing, sections were incubated with HRP‐conjugated secondary antibodies for 1 h. Signals were visualized with DAB and counterstained with hematoxylin.

### 2.9. Animal Experiments

Female C57BL/6J mice (6 weeks old) were supplied by Beijing Vital River Laboratory Animal Technology Co., Ltd. and maintained under specific pathogen‐free (SPF) conditions with a 12‐h light/dark cycle and free access to food and water. Animal experiments were reported in accordance with the ARRIVE guidelines. All animals were randomly assigned to three groups (*n* = 5 per group): (1) NC (normal control, vehicle cream 50 mg/day), (2) VIT (vitiligo model, 40% monobenzone cream 50 mg/day), and (3) VIT + Flu (vitiligo + fludarabine, 40% monobenzone cream 50 mg/day + fludarabine 20 mg/kg/day intraperitoneally). Topical application of monobenzone or vehicle cream was performed daily for 8 weeks. In the VIT + Flu group, fludarabine treatment was initiated at Week 5 and continued until the end of the experiment. Skin depigmentation was assessed weekly. At the endpoint, mice were anesthetized via intraperitoneal injection of 2% tribromoethanol (250 mg/kg) and euthanized by exsanguination under deep anesthesia to collect tissue samples. All experimental procedures were approved by the Institutional Animal Care and Use Committee of Beijing University of Chinese Medicine (Approval Number BUCM‐2024110103‐4077).

### 2.10. Cell Culture and Treatments

The murine macrophage cell line RAW264.7 was purchased from Cell Bank of the Chinese Academy of Sciences (Shanghai, China). RAW264.7 cells were maintained in RPMI‐1640 medium supplemented with 10% fetal bovine serum and 1% penicillin–streptomycin at 37°C with 5% CO_2_. For M1 macrophage induction, cells were seeded in 6‐well plates and treated with LPS (100 ng/mL, Sigma‐Aldrich, *Escherichia coli serotype O55:B5* lipopolysaccharide), fludarabine (10 μM, MCE), or both for 24 h. Cells were collected for protein or RNA analysis as indicated.

### 2.11. Enzyme‐Linked Immunosorbent Assay (ELISA)

TNF‐*α* concentrations in culture supernatants were measured using a mouse TNF‐*α* ELISA kit (EK282/3, LiankeBio) according to the manufacturer’s instructions. After 24 h treatment, supernatants were collected, centrifuged (1000 × *g*, 10 min, 4°C), and stored at −80°C. Standards and samples were added to antibody‐coated plates, incubated, and sequentially treated with detection antibody and streptavidin–HRP. TMB substrate was added, and reactions were stopped with 2MH_2_SO_4_. Absorbance was read at 450 nm (Bio‐Rad, USA), and TNF‐*α* concentrations were calculated from a standard curve.

### 2.12. Flow Cytometry Analysis

RAW264.7 cells were harvested after treatment, washed with PBS, and resuspended in FACS buffer (PBS + 2% FBS). Cells were stained with APC‐conjugated anti‐mouse CD86 antibody (1:200, Cat# 757943, BD Bioscience) for 30 min at 4°C in the dark, washed, and resuspended in buffer. Samples were analyzed on a BD FACSCanto II flow cytometer, and data were processed using FlowJo (v10.8.1). The proportion of CD86^+^ cells was calculated for each group.

### 2.13. Statistical Analysis

All statistical analyses were conducted in R (v4.2.0). Wilcoxon rank‐sum or *t* tests were used for two‐group comparisons. Multiple testing correction was performed using the Benjamini–Hochberg method. For comparisons of gene expression levels or pathway scores across multiple macrophage subpopulations, nonparametric statistical testing was performed using the Kruskal–Wallis one‐way analysis of variance (implemented as kruskal.test in R). Statistical significance was defined as *p* < 0.05 unless otherwise specified.

## 3. Results

### 3.1. Single‐Cell Transcriptomic Profiling Reveals Altered Cellular Landscape in Vitiligo Skin

To comprehensively characterize the cellular heterogeneity and molecular mechanisms underlying vitiligo pathogenesis, we reanalyzed a public scRNA‐seq data on skin samples from both healthy controls (HCs) and vitiligo patients. After quality control and filtering, we obtained high‐quality transcriptomic profiles from 48,506 cells across both conditions. UMAP dimensionality reduction revealed distinct cellular clusters, which were annotated into nine major cell types based on canonical marker gene expression (Figure 1A,B). These included MELs, KRTs, CD8^+^ T cells (CD8^+^TCs), macrophages and dendritic cells (MACs‐DCs), CD4^+^ T cells (CD4^+^TCs), LCs, FIBs, ECs, and SMCs. Cell‐type classification was validated by the expression of well‐established marker genes. Specifically, KRTs expressed high levels of *KRT1*, *KRT10*, *KRT14*, and *KRT15*, while MELs were marked by *DCT*, *PMEL*, *TYRP1*, and *MLANA*. FIBs were characterized by *COL1A1*, *DCN*, *SFRP2*, and *TWIST2*, and ECs by *PECAM1*, *CLEC14A*, *AQP1*, and ***ECSCR***. SMCs expressed *TAGLN*, *ACTA2*, *MYL9*, and *NR2F2*. CD4^+^ TCs were defined by *CD3D*, *CD3E*, *LTB*, *IL7R*, and *CD4*, while CD8^+^ TCs expressed *CD3D*, *CD3E*, *CD8A*, and *CD8B*. LCs were identified by *CD207*, *CD1A*, *FCGBP*, and *S100B*, and MAC‐DCs by *LYZ*, *CD1C*, *IL1B*, and *CLEC10A* (Figure 1B).

Figure 1Single‐cell RNA sequencing analysis reveals distinct cellular composition between healthy control and vitiligo skin tissues. (A) UMAP visualization of single‐cell RNA sequencing data from healthy control (HC) and vitiligo skin samples. Each point represents an individual cell, colored by cell type. Pie charts indicate the proportion of cells from HC (green) and vitiligo (red) samples for each cell type. Cell types include MELs (melanocytes), KRTs (keratinocytes), CD8^+^TC (CD8^+^ T cells), MACs (macrophages), CD4^+^TC (CD4^+^ T cells), LCs (langerhans cells), FIBs (fibroblasts), ECs (endothelial cells), and SMCs (smooth muscle cells). (B) Dot plot showing the expression of canonical marker genes across different cell types. Dot size represents the percentage of cells expressing each gene, and color intensity indicates the average expression level. Key marker genes are displayed for each cell type to validate the cellular identity. (C) Stacked bar chart comparing the cellular composition between HC and vitiligo samples. The chart shows the relative proportion of each cell type, highlighting differences in cellular distribution between the two conditions. (D) Ro/e (observed/expected ratio) index heatmap illustrating the enrichment or depletion of specific cell types in vitiligo compared to HC samples. Values above 1 (red) indicate enrichment in the respective condition, while values below 1 (green) suggest depletion. Numbers within each cell represent the Ro/e index values.

**Figure:**
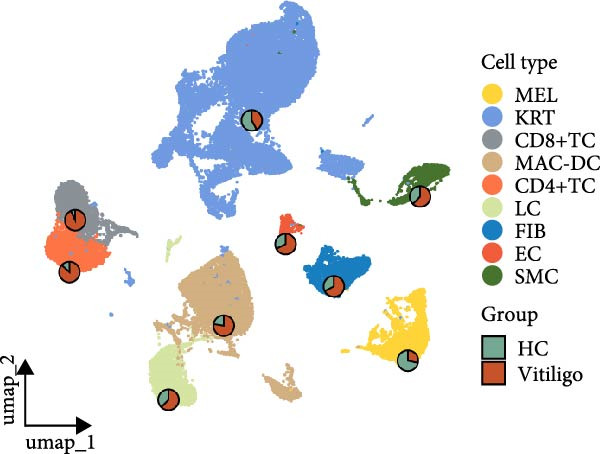
(A)

**Figure:**
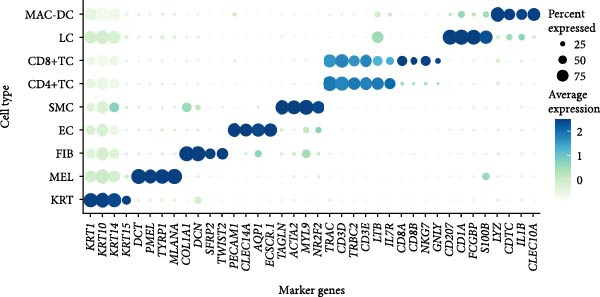
(B)

**Figure:**
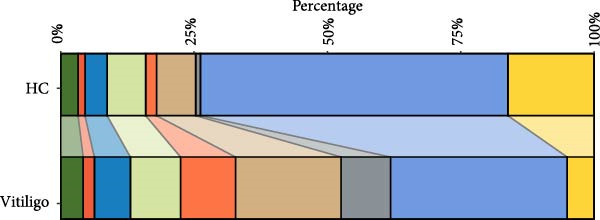
(C)

**Figure:**
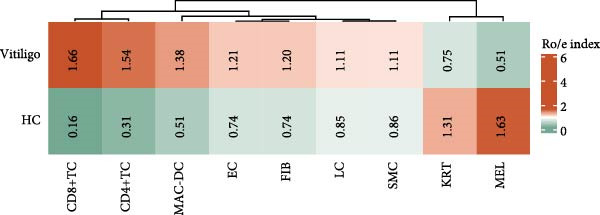
(D)

Comparative analysis of cell composition between HCs and vitiligo samples revealed marked changes in the skin microenvironment (Figure 1C). Vitiligo skin showed a significant loss of MELs, consistent with pigment depletion, and an increased proportion of immune cells, particularly MAC‐DCs and T cell subsets, indicative of an inflammatory milieu. To quantify these shifts, we calculated the Ro/e ratio for each cell type (Figure 1D), confirming MEL depletion (Ro/e < 1) and enrichment of macrophages, CD4^+^, and CD8^+^ TCs (Ro/e > 1). These findings lay the groundwork for subsequent investigation into macrophage subtypes and their roles in vitiligo pathogenesis.

### 3.2. Macrophage Heterogeneity Analysis Reveals Distinct Inflammatory Subpopulation in Vitiligo

Given the observed enrichment of macrophages in vitiligo skin that was not fully explored in vitiligo pathology, we performed detailed subcluster analysis of macrophage populations to characterize their heterogeneity and functional states. Reclustering of the MAC‐DC compartment revealed five transcriptionally distinct macrophage subpopulations—immunoregulatory macrophages (Mac‐IR), ribosome‐enriched macrophages (Mac‐Ribo), tissue‐remodeling macrophages (Mac‐TR), oxidative phosphorylation–activated macrophages (Mac‐OxP), and Mac‐InflamAP—as well as three DC subsets, including proliferating DCs (DC‐Prolif), conventional DCs (cDCs), and plasmacytoid DCs (pDCs) (Figure 2A). Each subset exhibited a distinct marker gene expression profile, supporting its unique functional identity (Figure 2B, Supporting Information 4: Figure S1A).

Figure 2Macrophage subpopulation analysis and pseudotime trajectory reveal Mac‐InflamAP as a terminal inflammatory state in vitiligo. (A) UMAP visualization of macrophage subpopulations identified through reclustering analysis. Each point represents a macrophage cell, colored by subtype: immunoregulatory macrophages (Mac‐IR), ribosome‐enriched macrophages (Mac‐Ribo), tissue‐remodeling macrophages (Mac‐TR), proliferating macrophages (Mac‐Prolif), oxidative phosphorylation–activated macrophages (Mac‐OxP), and inflammatory antigen‐presenting macrophages (Mac‐InflamAP). Pie charts show the proportion of cells from HC (green) and vitiligo (red) samples for each subpopulation. (B) Dot plot displaying the expression of marker genes across macrophage subpopulations. Dot size represents the percentage of cells expressing each gene, and color intensity indicates the normalized expression level (*z*‐score). Upper pie charts show the tissue source distribution for each subpopulation. (C) Heatmap showing the Ro/e (observed/expected ratio) index for each macrophage subpopulation in HC and vitiligo samples. Values represent the relative enrichment (red, >1) or depletion (green, <1) of each subpopulation in the respective conditions. (D) Violin plots comparing M1 and M2 macrophage polarization scores across different macrophage subpopulations. (E) Violin plots comparing potency scores calculated by CytoTRACE2 across different macrophage subpopulations. (F) Pseudotime trajectory analysis of macrophage subpopulations. Left panel shows cells colored by subtype, and right panel shows pseudotime progression from early (purple) to late (yellow) stages. Black circles with numbers indicate trajectory branch points. Cells in gray represent small or spatially disconnected clusters that were excluded from the trajectory calculation. These cells are included in the UMAP embedding but are not assigned pseudotime values. (G) Heatmap showing pseudotime‐dependent gene expression patterns across the trajectory. Genes are clustered into distinct temporal expression patterns, with Cluster 1 and 2 specifically enriched in Mac‐InflamAP cells. Cell metadata includes subtype, pseudotime, and tissue source information. (H and I) Gene Ontology (GO) enrichment analysis for Cluster 1 (H) and Cluster 2 (I) genes. Dot plots show the top 20 significantly enriched biological processes, with colors indicating *p*‐value of enrichment. *p*‐Values in violin plots (D and E) were calculated using the Kruskal–Wallis one‐way nonparametric test, representing overall (omnibus) significance across all compared macrophage subpopulations.

**Figure:**
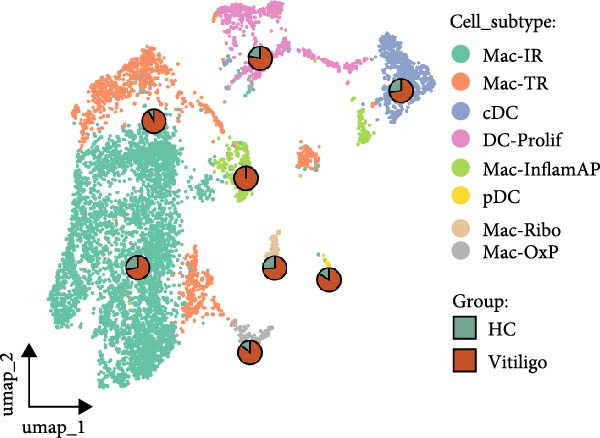
(A)

**Figure:**
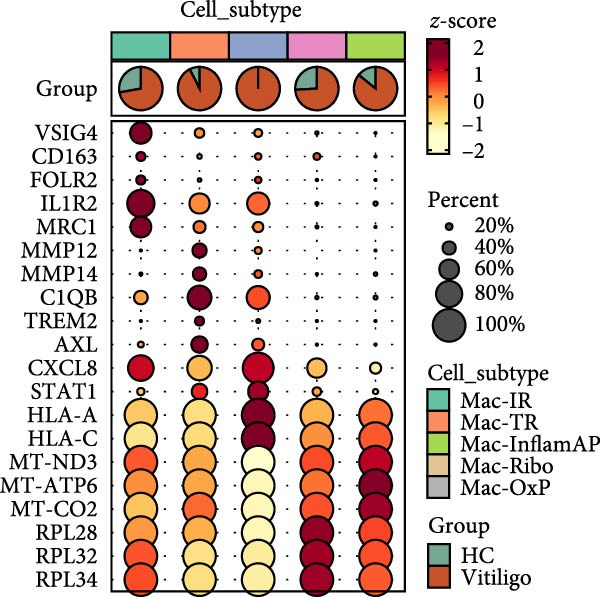
(B)

**Figure:**
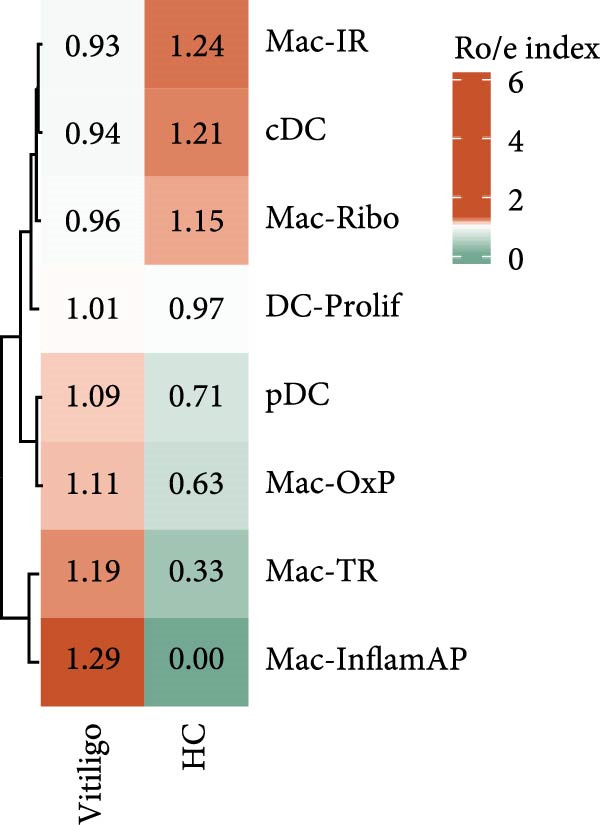
(C)

**Figure:**
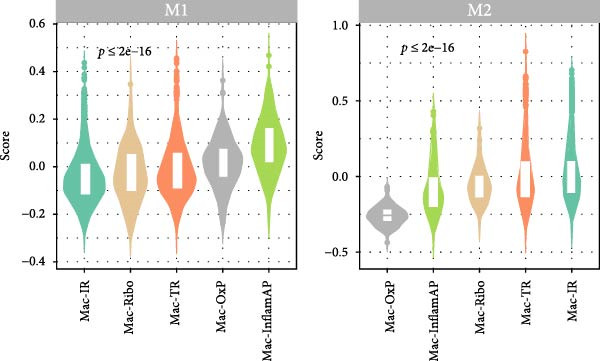
(D)

**Figure:**
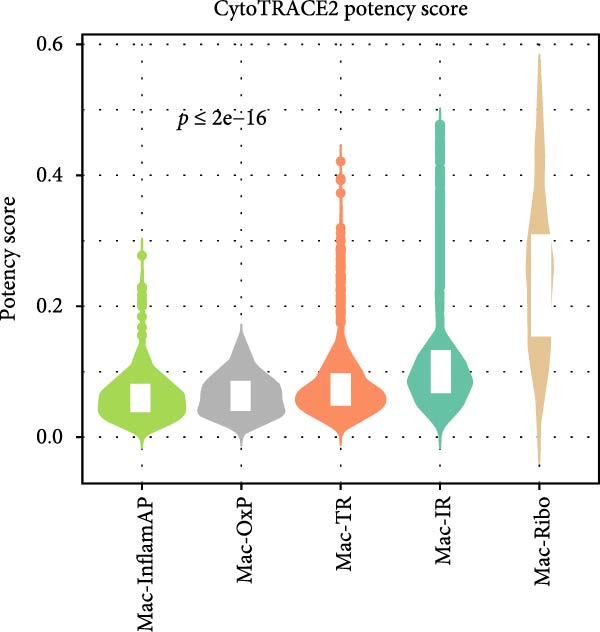
(E)

**Figure:**
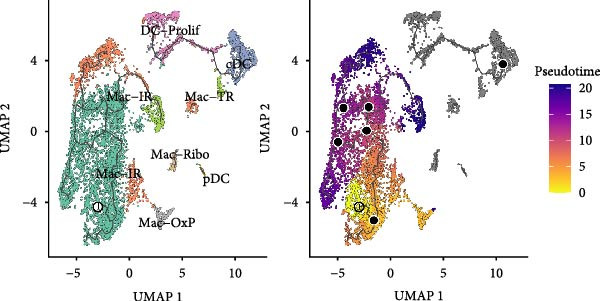
(F)

**Figure:**
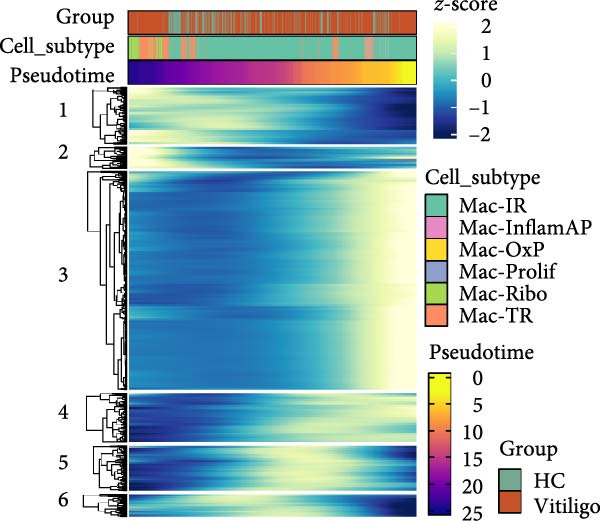
(G)

**Figure:**
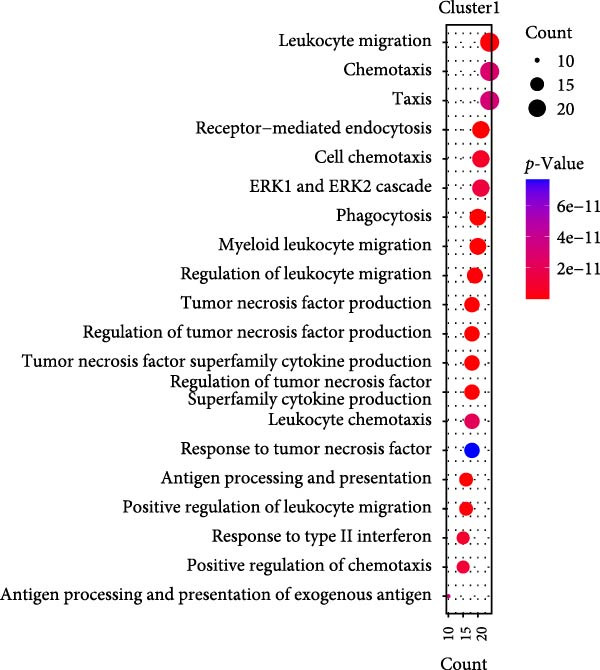
(H)

**Figure:**
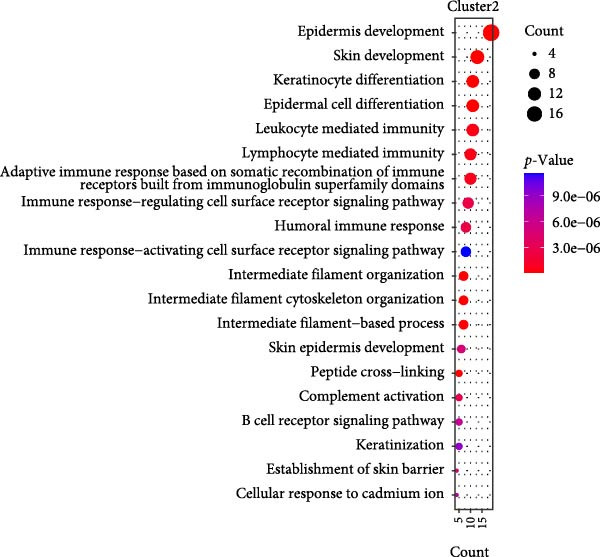
(I)

Comparative analysis of macrophage subpopulation distribution between HC and vitiligo samples revealed striking differences in their representation (Figure 2C). Most notably, the Mac‐InflamAP subpopulation showed dramatic enrichment in vitiligo samples (Ro/e = 1.29), while being nearly absent in HCs (Ro/e = 0.00). This finding suggests that Mac‐InflamAP represents a pathological macrophage state specifically associated with vitiligo pathogenesis. Consistently, Mac‐TR showed relative depletion in HC (Ro/e = 0.33 vs. 1.19 in vitiligo), indicating disrupted tissue homeostasis mechanisms.

To validate the robustness and reproducibility of macrophage subcluster identification, we further analyzed an independent scRNA‐seq dataset (GEO: GSE288871) comprising skin samples from both HCs and vitiligo patients (Supporting Information 4: Figure S1B,C). Following integration and batch correction with the primary dataset, we successfully reproduced all five macrophage subtypes (Mac‐IR, Mac‐Ribo, Mac‐TR, Mac‐OxP, and Mac‐InflamAP) with consistent marker gene expression profiles (Supporting Information 4: Figure S1D,E). Cross‐dataset correlation analysis demonstrated high transcriptional concordance between corresponding macrophage subsets across the two cohorts (Pearson’s *r* = 0.68–0.80, Supporting Information 4: Figure S1F), underscoring the robustness and generalizability of the macrophage subtype definitions.

To further characterize the polarization states of these macrophage subpopulations, we assessed M1 and M2 macrophage signature scores across all subtypes (Figure 2D). Mac‐InflamAP exhibited the highest M1 polarization score (*p*  < 2e‐16), consistent with its proinflammatory phenotype, while showing reduced M2 characteristics. In contrast, Mac‐IR and Mac‐TR displayed higher M2 signatures, suggesting their involvement in tissue repair and immune regulation processes.

### 3.3. Pseudotime Trajectory Analysis Reveals Mac‐InflamAP as a Terminal Differentiation State

To understand the developmental relationships and temporal dynamics among macrophage subpopulations, we performed pseudotime trajectory analysis using CyotTRACE2 and Monocle3. According to CytoTRACE2 that was a computational method for predicting cellular potency categories and absolute developmental potential from scRNA‐seq data, Mac‐InflamAP exhibited lowest potency score across the Mac subtypes (Figure 2E), suggesting that Mac‐InflamAP represents a late stage differentiation state, potentially arising from the differentiation of other macrophage subpopulations under inflammatory conditions.

We further employed Monocle3 to delineate the complex differentiation trajectory with multiple branching pathways. The parameter use_partition = TRUE was set to minimize the influence of spatial discontinuity and small, disconnected clusters on the inferred trajectories, particularly for DCs and Mac‐Ribo subsets. Integrating these findings with CytoTRACE2 analysis, we designated Mac‐IR cells as the starting point of differentiation. Consistently, Monocle3 revealed that Mac‐InflamAP cells predominantly occupied the terminal regions along the pseudotime trajectory, a result that was in strong agreement with the CytoTRACE2 inference (Figure 2E,F). Pseudotime‐dependent gene expression analysis identified distinct gene clusters that were dynamically regulated along the trajectory (Figure 2G). Notably, two gene clusters (Cluster 1 and Cluster 2) showed specific enrichment in Mac‐InflamAP cells, indicating unique transcriptional programs associated with this inflammatory subpopulation. Cluster 1 genes exhibited early upregulation followed by sustained high expression, while Cluster 2 genes showed progressive upregulation toward the terminal pseudotime states. GO enrichment analysis of these clusters revealed distinct functional themes (Figure 2H,I, Supporting Information 4: Figure S1G,H and Supporting Information 2: Table S2). Cluster 1 genes were significantly enriched in pathways related to antigen processing and presentation, phagocytosis, and tumor necrosis factor production, highlighting the antigen‐presenting and proinflammatory capabilities of Mac‐InflamAP (Figure 2H and Supporting Information 4: Figure S1G). Cluster 2 genes showed enrichment in pathways associated with epidermal development, KRT differentiation, and skin development, suggesting complex interactions between Mac‐InflamAP and skin structural components (Figure 2I and Supporting Information 4: Figure S1H). Additionally, both clusters showed enrichment in immunoglobulin‐related processes, indicating enhanced antigen presentation capacity that may contribute to autoimmune responses in vitiligo.

### 3.4. Cell–cell Communication Analysis Reveals Pathological Interactions Mediated by Mac‐InflamAP

To understand the functional impact of macrophage subpopulations in vitiligo pathogenesis, we performed comprehensive cell–cell communication analysis using CellChat. Overall communication analysis revealed a substantial increase in the total number of interactions in vitiligo samples compared to HCs (3940 vs 2473) (Figure 3A), with interaction strength also showing marked elevation (0.08 vs. 0.037). This global increase in cellular communication suggests a highly active and dysregulated signaling environment in vitiligo‐affected skin. Two‐dimensional visualization of cell–cell communication networks highlighted the central role of macrophages, particularly the Mac‐InflamAP subpopulation, in mediating intercellular signaling in vitiligo (Figure 3B, Supporting Information 4: Figure S2A,B). The alternative interaction number and strength between HC and vitiligo revealed that Mac‐InflamAP occupied a critical hub position, establishing extensive connections with multiple cell types including T cells, MELs, and other stromal and immune cells (Supporting Information 4: Figure S2A,B), indicating its pivotal role in orchestrating the pathological immune response.

Figure 3Cell–cell communication analysis reveals pathological signaling networks mediated by Mac‐InflamAP in vitiligo. (A) Bar plots comparing the total number of interactions (A1) and interaction strength (A2) between healthy control (HC) and vitiligo samples. Values represent the overall cellular communication activity in each condition. (B) Two‐dimensional scatter plots visualizing cell–cell communication networks in HC (B1) and vitiligo (B2) samples. Each point represents a cell type, with position determined by outgoing and incoming interaction strength. Point size indicates the total number of interactions and colors represent different cell types. (C) Stacked bar chart showing the relative activity of signaling pathways in HC (green) and vitiligo (red) samples. Each bar represents a specific signaling pathway, with the proportion indicating the contribution of each condition to the total pathway activity. (D) Dot plot illustrating the communication probabilities from Mac‐InflamAP to CD8^+^T cells in HC and vitiligo samples. Each column represents a distinct signaling pathway, with color intensity reflecting the probability of interaction. Pathways with significant enrichment (*p* < 0.05) are indicated by colored dots. (E) Dot plot showing the communication probabilities from Mac‐InflamAP to melanocytes (MELs) in HC and vitiligo samples. Notably, the analysis highlights TNF and other cytotoxic signaling pathways that may drive MEL destruction in vitiligo. (F) Dot plot depicting the communication probabilities from CD4/8^+^T cells to Mac‐InflamAP in HC and vitiligo samples, revealing altered immune–macrophage interactions in disease states. (G) Violin plot comparing TNF signaling pathway scores across different macrophage subpopulations. (H) Violin plot comparing MHC‐I and MHC‐II pathway scores across macrophage subpopulations. Both pathways show significantly higher expression in Mac‐InflamAP (*p*  < 2e‐16), indicating enhanced antigen presentation capacity. *p*‐Values in violin plots (G and H) were calculated using the Kruskal–Wallis one‐way nonparametric test, representing overall (omnibus) significance across all compared macrophage subpopulations.

**Figure:**
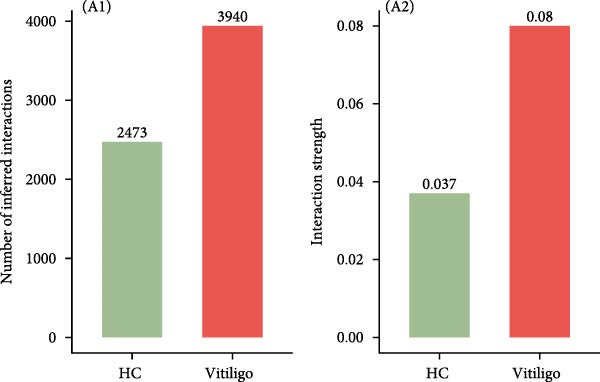
(A)

**Figure:**
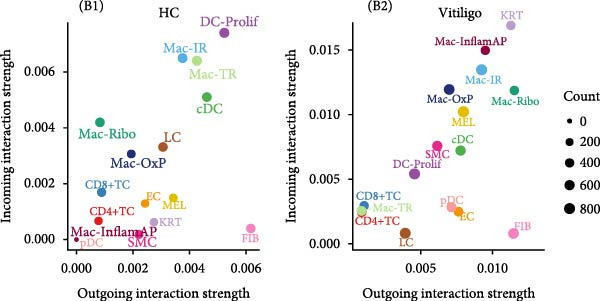
(B)

**Figure:**
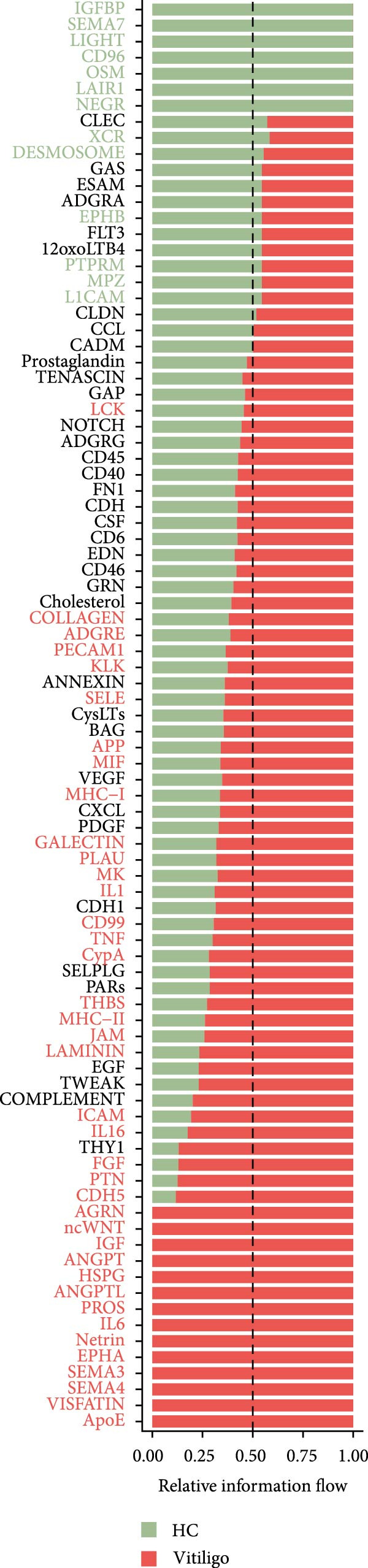
(C)

**Figure:**
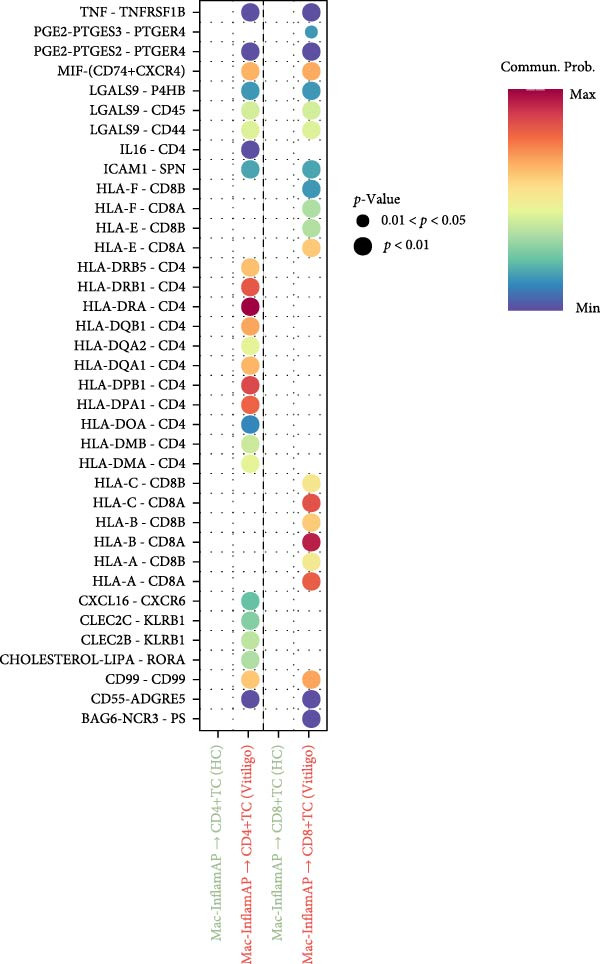
(D)

**Figure:**
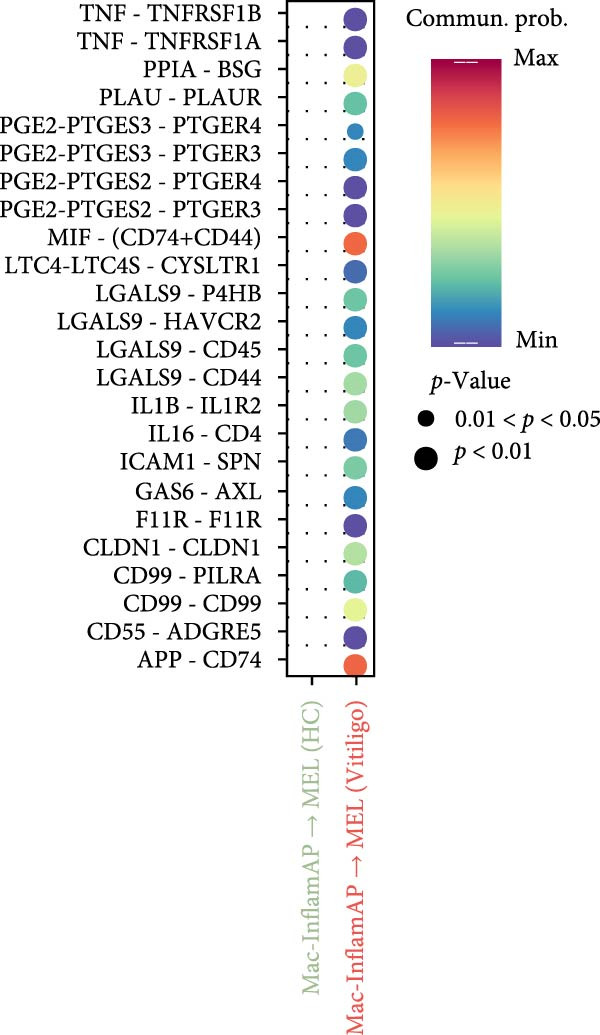
(E)

**Figure:**
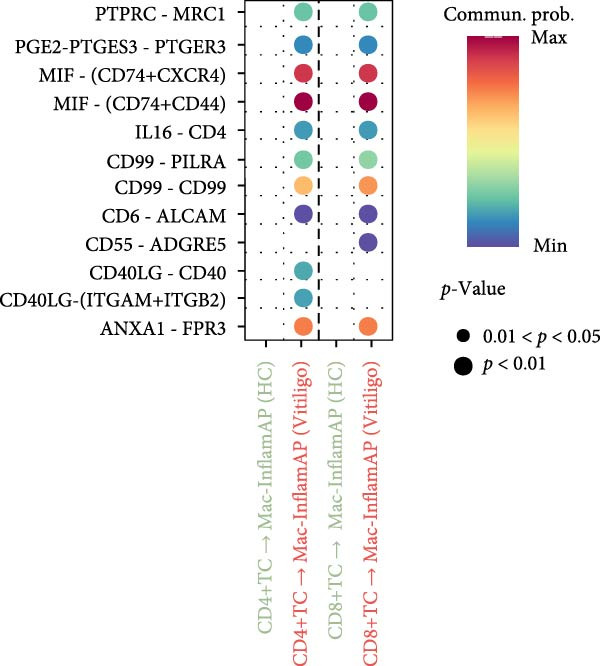
(F)

**Figure:**
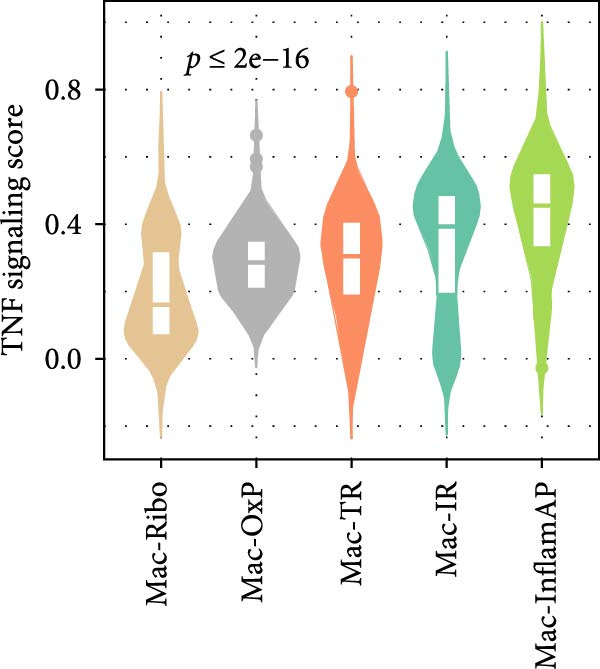
(G)

**Figure:**
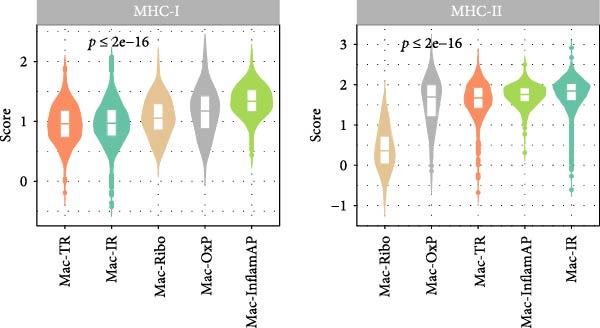
(H)

Comparative analysis of signaling pathways between vitiligo and HC samples identified numerous differentially regulated communication pathways (Figure 3C). Several inflammatory and immune‐related pathways showed significant upregulation in vitiligo, including IL1, IL6, TNF, MHC‐I, MHC‐II, and various cytokine signaling cascades. Conversely, pathways associated with tissue homeostasis and regeneration were enriched in HC, including CD96 and IGFBP, reflecting the disrupted balance between inflammation and repair in vitiligo skin.

Focused analysis of Mac‐InflamAP‐mediated communications revealed distinct interaction patterns with key cell types involved in vitiligo pathogenesis (Figure 3D–F). Mac‐InflamAP showed enhanced outgoing signaling to CD4^+^ T cells and CD8^+^ T cells in vitiligo samples, particularly through MHCII and MHC‐I and costimulatory pathways (Figure 3D), suggesting its role in promoting T cell activation. Most notably, Mac‐InflamAP demonstrated significantly increased TNF signaling to MELs in vitiligo (Figure 3E), which is consistent with the known cytotoxic effects of TNF‐*α* on pigment‐producing cells. Additionally, CD4^+^ TCs and CD8^+^ TCs in vitiligo exhibited an enhanced communication to Mac‐InflamAP that regulated antigen presentation (PTPRC ‐ MRC1), chemokine (MIF signaling), inflammation (IL16 ‐ CD4), and T cell costimulation (CD6 ‐ ALCAM and CD55 ‐ ADGRE5), indicating a positive feedback loop that may perpetuate the inflammatory response.

To validate these communication patterns, we assessed the expression of key signaling pathway components across macrophage subpopulations. TNF signaling pathway scores were significantly elevated in Mac‐InflamAP compared to other subpopulations (*p*  < 2e‐16, Figure 3G), confirming its enhanced capacity for TNF‐mediated cytotoxicity toward MELs. Similarly, MHC‐I and MHC‐II pathway scores showed the highest expression in Mac‐InflamAP (*p*  < 2e‐16, Figure 3H), supporting its prominent role in antigen presentation and T cell activation. These findings collectively demonstrate that Mac‐InflamAP functions as a central mediator of pathological cell–cell communication in vitiligo, promoting both MEL destruction through TNF signaling and autoimmune responses through enhanced antigen presentation.

Moreover, we also observed alternative cell–cell interactions between HC and vitiligo conditions. Specifically, LC and Mac‐TR demonstrated enhanced MHC‐I and CD4 signaling with CD4^+^ T cells (Supporting Information 4: Figure S2C,D), indicating alternatively enhanced antigen presentation in vitiligo skin. Mac‐TR was also participating in collagen CD44/SCD4 signaling with MEL (Supporting Information 4: Figure S2E). MEL from vitiligo skins also exhibited an enhanced MHC‐I and CD4 singaling with CD4^+^ T cells compared to HCs (Supporting Information 4: Figure S2F), indicating intrinsic alternation of MEL in vitiligo pathology.

### 3.5. hdWGCNA Identifies Key Regulatory Modules and Therapeutic Targets in Macrophages

To elucidate the regulatory programs underlying macrophage dysfunction in vitiligo, we performed hdWGCNA specifically on macrophage populations. This approach constructed co‐expression modules reflecting functionally related gene sets that modulate macrophage behavior under different conditions. A soft threshold of *β* = 8 was selected to ensure scale‐free topology (*R* ^2^ > 0.8) and optimal network connectivity (Supporting Information 4: Figure S3A). In total, 15 distinct coexpression modules (Mac‐M1 to Mac‐M15) were identified through hierarchical clustering (Figure 4A). Module–module correlation analysis revealed complex relationships, with Mac‐M1, M2, M3, M4, M9, M11, and M12 showing positive correlations, suggesting coordinated but distinct regulatory programs (Figure 4B). Cell type‐specific module scoring revealed that several modules—particularly Mac‐M1, M2, M3, M4, M9, M11, M12, and M13—were predominantly active in macrophages andLCs (Figure 4C). The genes comprising each coexpression module, along with their corresponding module membership scores, are provided in Supporting Information 3: Table S3.

Figure 4hdWGCNA identifies key regulatory modules and potential therapeutic targets in macrophages. (A) Hierarchical clustering dendrogram showing the construction of coexpression modules in macrophages. The dendrogram displays gene clustering patterns, with different colors at the bottom representing distinct modules (Mac‐M1 to Mac‐M14). The height indicates the dissimilarity between gene clusters. (B) Module–module correlation heatmap displaying the relationships between different coexpression modules. Color intensity represents the correlation strength, with red indicating positive correlations and blue indicating negative correlations. Nonsignificant correlations are marked with an “x.” (C) Dot plot showing module gene scores across different cell types. Dot size represents the percentage of cells expressing module genes, and color intensity indicates the average expression level. This analysis validates the cell‐typespecificity of identified modules. (D) Lollipop plot comparing module activities between vitiligo and HC macrophages. The *x*‐axis shows the average log_2_ fold change for each module, with colors representing different modules. Mac‐M3 shows the highest upregulation in vitiligo samples. Nonsignificant correlations are marked with an “x.” (E) Protein–protein interaction (PPI) network of Mac‐M3 module genes. Each node represents a gene, with node size proportional to its degree within the network. Edge thickness reflects the strength of protein interactions. The network demonstrates the interconnected nature of module genes. (F) Bar plot showing the PPI degree (number of connections) for the top hub genes in the Mac‐M3 module. Higher degree values indicate more central positions in the network, suggesting greater regulatory importance. (G) Scatter plot integrating module membership (kME, *x*‐axis) with differential expression (log_2_ fold change, *y*‐axis) for Mac‐M3 module genes. The four prioritized therapeutic targets (STAT1, VAMP5, LAP3, and TYMP) are highlighted in red, representing genes with both high module membership and significant differential expression. (H) Violin plot comparing Mac‐M3 module scores across different macrophage subpopulations. Statistical significance was assessed using Wilcoxon rank‐sum test. Mac‐InflamAP was employed as reference group in the comparison.  ^∗∗∗∗^ *p*  < 0.0001. (I) Violin plots showing the expression levels of four prioritized target genes (STAT1, LAP3, VAMP5, and TYMP) across macrophage subpopulations. Statistical significance was assessed using Wilcoxon rank‐sum test. Mac‐InflamAP was employed as reference group in the comparison.  ^∗∗∗∗^ *p*  < 0.0001, ns, not significant.

**Figure:**
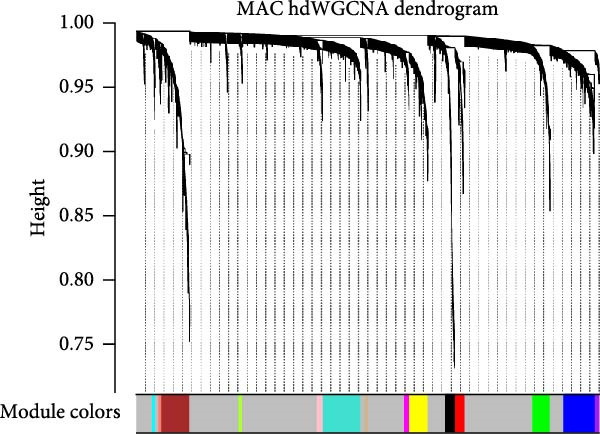
(A)

**Figure:**
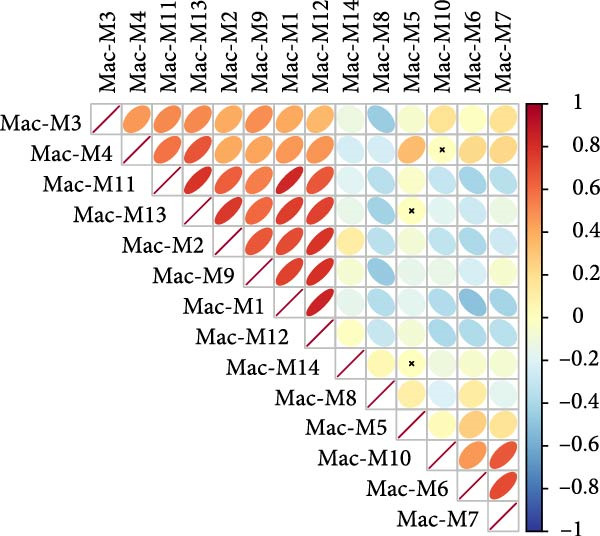
(B)

**Figure:**
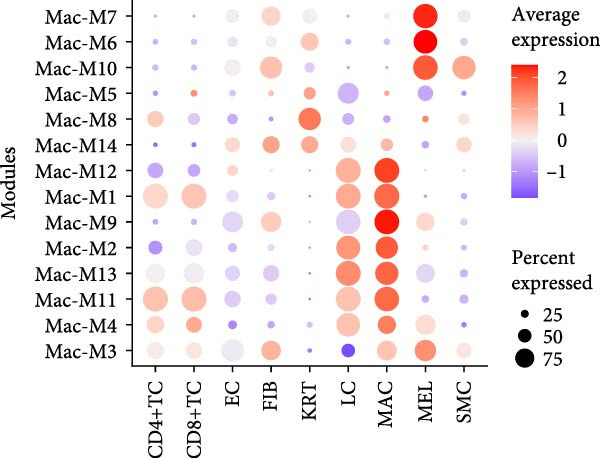
(C)

**Figure:**
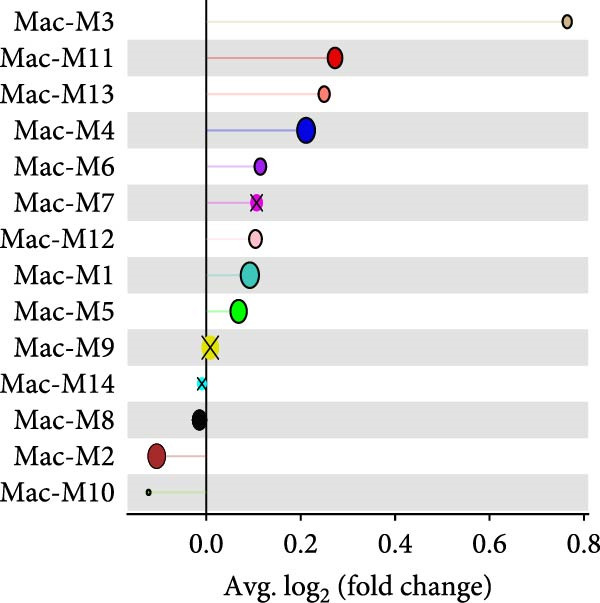
(D)

**Figure:**
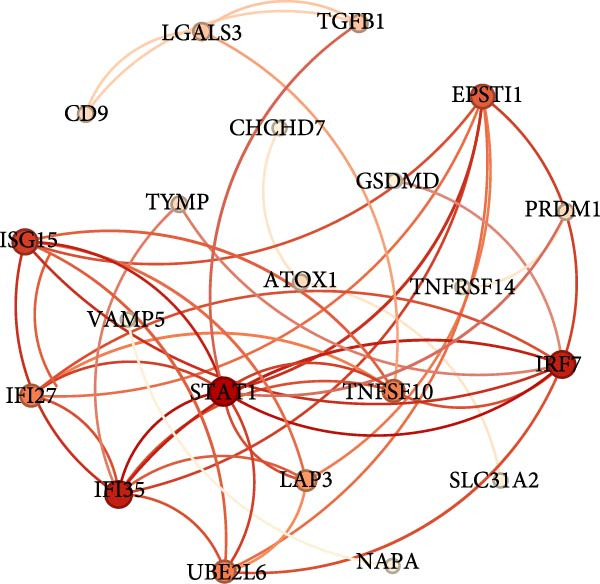
(E)

**Figure:**
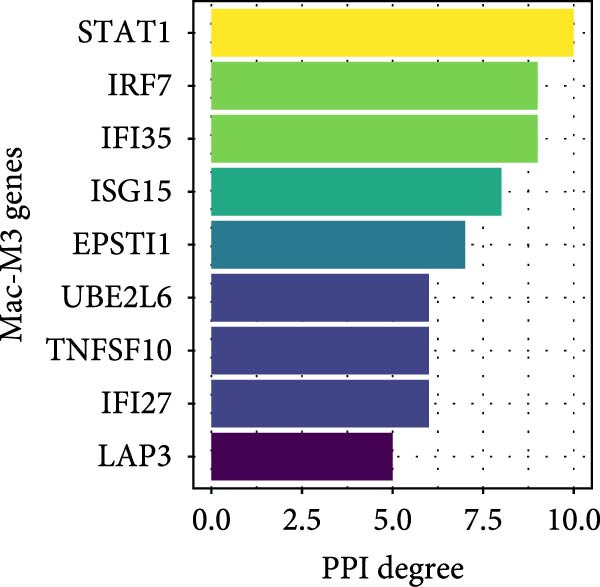
(F)

**Figure:**
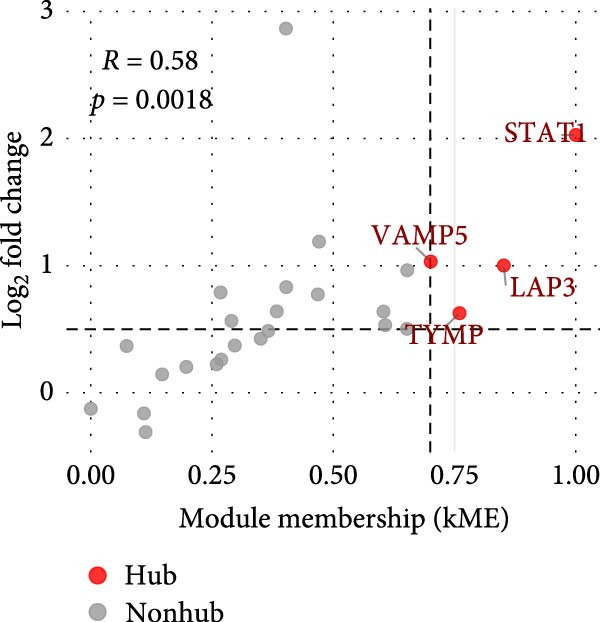
(G)

**Figure:**
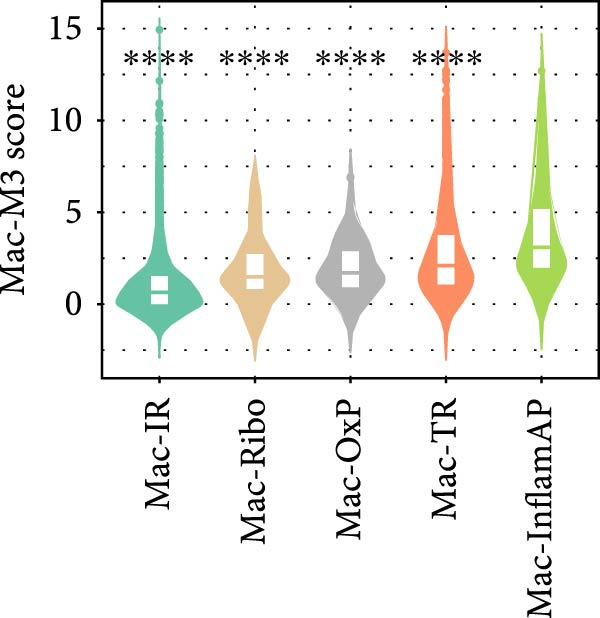
(H)

**Figure:**
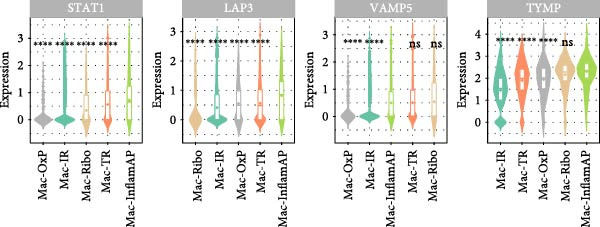
(I)

Among them, Mac‐M3 was the most significantly upregulated in vitiligo samples compared to HCs (Figure 4D). Notably, Mac‐M3 module activity was specifically elevated in the Mac‐InflamAP subpopulation (Figure 4H; Supporting Information 4: Figure S3B; *p*  < 2e‐16), linking this module directly to the inflammatory macrophage state enriched in vitiligo skin. This spatial and quantitative specificity highlights Mac‐M3 as a key module involved in disease‐associated macrophage reprogramming.

To understand the functional implications of Mac‐M3, we conducted GO and pathway analysis, revealing enrichment in IFN signaling, immune response, and myeloid cell migration and differentiation (Supporting Information 4: Figure S3C). PPInetwork analysis of Mac‐M3 genes showed a dense interaction network, with STAT1, IRF7, and IFIT27 emerging as highly connected hub genes (Figure 4E,F; Supporting Information 4: Figure S3D), indicating their central role in mediating inflammatory responses.

To prioritize therapeutic candidates, we integrated network connectivity (kME) with differential gene expression. This analysis identified four genes—*STAT1*, *VAMP5*, *LAP3*, and *TYMP*—that exhibited both strong module membership and significant upregulation in vitiligo (Figure 4G). All four were also significantly overexpressed in Mac‐InflamAP (Figure 4I), reinforcing their potential as core regulators of the pathogenic macrophage phenotype and as promising targets for therapeutic intervention.

### 3.6. STAT1 Suppression Inhibits Pathological Features of Vitiligo In Vivo

Given that STAT1 showed the strongest significance in PPI degree, module membership (kME), and differential expression—and was specifically enriched in the vitiligo‐associated Mac‐InflamAP population—we next validated its role in vivo by assessing the effects of STAT1 suppression on vitiligo pathology. Monobenzone was employed to induce vitiligo mice (VIT) while fludarabine was used as STAT1 inhibitor and served as the treatment group (VIT + Flu) (Figure 5A). Macroscopic evaluation revealed that fludarabine treatment significantly reduced depigmentation severity compared to the untreated model group, suggesting therapeutic efficacy in mitigating vitiligo progression (Figure 5B). Histological analysis with H&E staining showed that monobenzone induction led to marked epidermal thicking, immune infiltration, and structural alterations characteristic of vitiligo lesions compared to controls, whereas fludarabine treatment significantly restored epidermal structure and reduced thickness and immune infiltration, indicating improved tissue integrity (Figure 5C).

Figure 5STAT1 inhibition by fludarabine ameliorates vitiligo progression in a monobenzone‐induced mouse model. (A) Experimental design: C57BL/6J female mice were treated with vehicle or 10% monobenzone cream (50 mg/day) for 8 weeks. Groups: NC (vehicle), VIT (monobenzone + vehicle i.p.), and VIT + Flu (monobenzone + fludarabine, 20 mg/kg/day i.p., weeks 5–8). (B) Representative dorsal skin images at weeks 0, 4, and 8 showing reduced depigmentation with fludarabine. (C) H&E staining of dorsal skin. Higher magnification demonstrates preserved epidermal thickness and reduced immune infiltration (arrows) in VIT + Flu. (D) Western blot of p‐STAT1, STAT1, Bax, cleaved‐Caspase3, Bcl‐2, and Caspase3, with *β*‐actin as control. Fludarabine suppressed STAT1 phosphorylation and apoptosis while enhancing Bcl‐2. (E) Immunohistochemistry of CD4^+^/CD8^+^ T cells, F4/80^+^ macrophages, and CD86^+^ M1 macrophages. Fludarabine markedly reduced immune cell infiltration and M1 polarization. (F) qRT‐PCR of macrophage markers. Fludarabine downregulated Cd86 and iNOS (M1) and upregulated Cd163 and Arg1 (M2). Data are mean ± SEM (*n* = 5), *t* test.  ^∗^ *p*  < 0.05,  ^∗∗^ *p*  < 0.01,  ^∗∗∗^ *p*  < 0.001. NC, normal control; VIT, vitiligo model; VIT + Flu, vitiligo + fludarabine.

**Figure:**
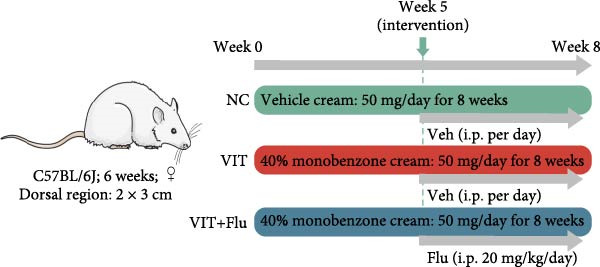
(A)

**Figure:**
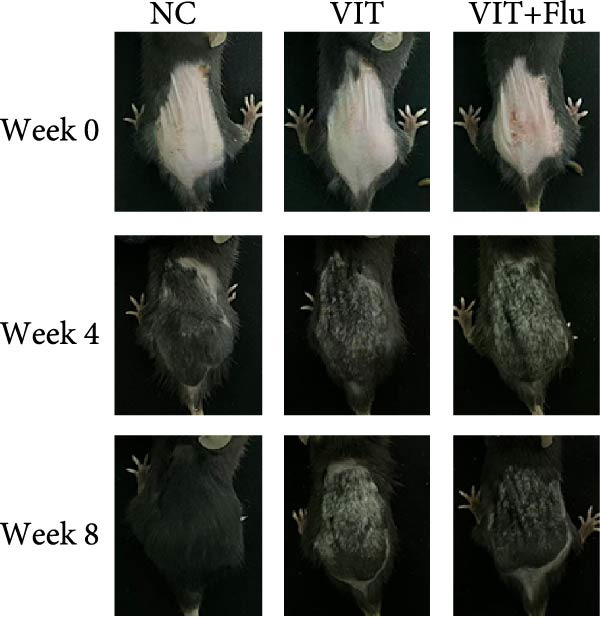
(B)

**Figure:**
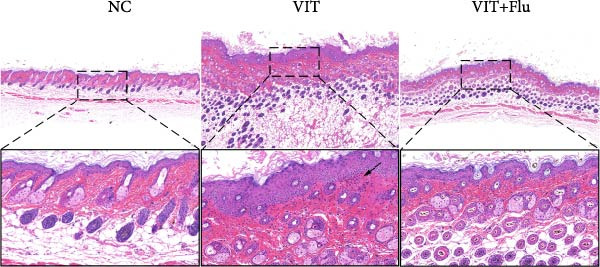
(C)

**Figure:**
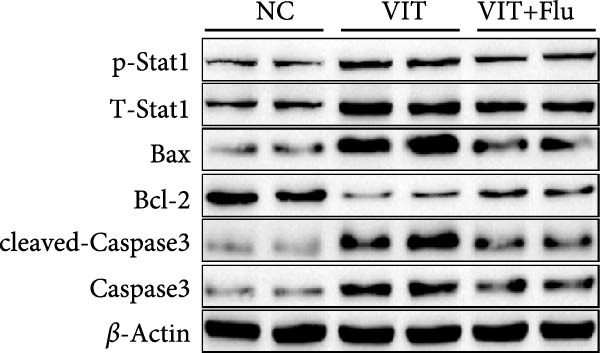
(D)

**Figure:**
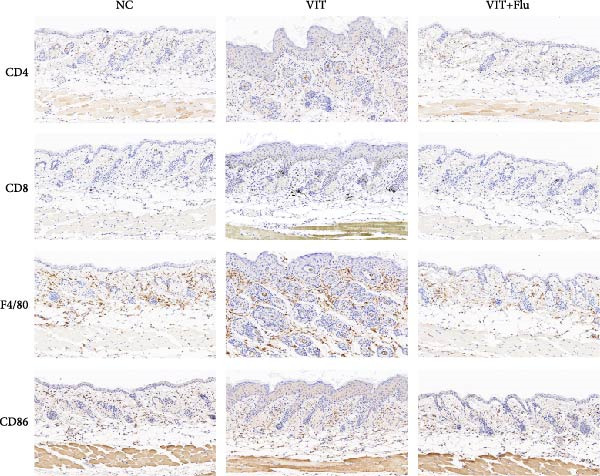
(E)

**Figure:**
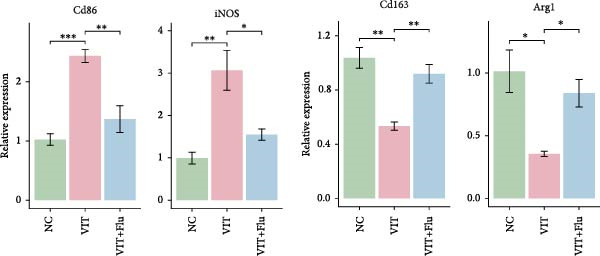
(F)

Immunohistochemical analysis identified changes in immune cell infiltration. CD4 and CD8 immunostaining revealed increased T cell infiltration in monobenzone‐induced lesions, which was significantly reduced by fludarabine treatment, indicating decreased immune activation (Figure 5D). Macrophage infiltration, assessed by F4/80 staining, was substantially elevated in monobenzone‐induced lesions relative to normal skin. Fludarabine significantly reduced macrophage accumulation (Figure 5D). Analysis of M1 macrophage marker (CD86) revealed a marked activation of M1 macrophage in the VIT group, while fludarabine treatment reduced M1 macrophage populations and maintained reduced melanogenic marker expression (Figure 5D). Advanced qRT‐PCR confirmed that fludarabine significantly reversed VIT‐induced M1 marker expression (Cd86, iNOS) and VIT‐inhibited M2 marker expression (Cd163, Arg1) (Figure 5E). Western blot analysis offered further mechanistic insights into STAT1 pathway inhibition. Fludarabine reduced both total STAT1 and phosphorylated STAT1 (p‐STAT1 Y701), confirming effective suppression of the STAT1 pathway (Figure 5F). Apoptosis markers (BAX, cleaved Caspase‐3) were reduced, while BCL‐2 expression increased, suggesting decreased cell death and enhanced survival (Figure 5F).

These findings demonstrate that fludarabine‐mediated STAT1 inhibition ameliorates vitiligo progression by suppressing macrophage‐driven inflammation, reducing T cell activity, preserving MELs, and promoting melanogenesis. The observed therapeutic effects provide compelling preclinical evidence supporting STAT1 as a promising target for vitiligo treatment.

### 3.7. STAT1 Inhibition in Macrophages Reduces Antigen Presentation and Inflammatory Phenotype In Vitro

To investigate the functional role of STAT1 in macrophage‐mediated vitiligo pathology, we conducted in vitro experiments using RAW264.7 murine macrophages. scRNA‐seq analysis indicated that the vitiligo‐enriched Mac‐InflamAP subset shares key molecular features with LPS‐stimulated macrophages, including heightened inflammatory activity, enhanced antigen presentation, and a prototypical M1‐like activation phenotype. Accordingly, we used LPS stimulation to mimic the activation state of Mac‐InflamAP cells and evaluated whether pharmacological inhibition of STAT1 with fludarabine could attenuate these pathological molecular alterations. Western blot analysis confirmed that fludarabine markedly suppressed total STAT1 as well as phosphorylation at Tyr701 in LPS‐stimulated macrophages (Figure 6A).

Figure 6Fludarabine suppresses LPS‐induced macrophage M1 activation and inflammatory responses in RAW264.7 cells. (A) Western blot showing p‐STAT1 and STAT1 levels, with *β*‐actin as control. (B) Flow cytometry histograms of CD86 expression. LPS increased fluorescence intensity, attenuated by fludarabine. (C) Quantification of CD86^+^ cells (%) illustrated by bar plot. LPS markedly increased CD86^+^ cells, which were reduced by fludarabine. (D) ELISA of TNF‐*α* in culture supernatants. Fludarabine significantly suppressed LPS‐induced cytokine release. (E) qRT‐PCR of antigen presentation‐related genes (Cd86, H2‐Aa, H2‐Ab1, H2‐D1, and H2‐K1). Fludarabine downregulated LPS‐induced expression. (F) qRT‐PCR of inflammatory cytokines (Il6, Il1*β*, Tnf*α*, Ifn*α*, and Ifn*γ*). Fludarabine significantly reduced LPS‐induced transcription. Data are mean ± SEM (*n* = 3), *t* test.  ^∗∗^ *p*  < 0.01,  ^∗∗∗^ *p*  < 0.001; Flu, fludarabine alone; LPS, lipopolysaccharide; LPS + Flu, LPS + fludarabine; NC, negative control; ns, not significant.

**Figure:**
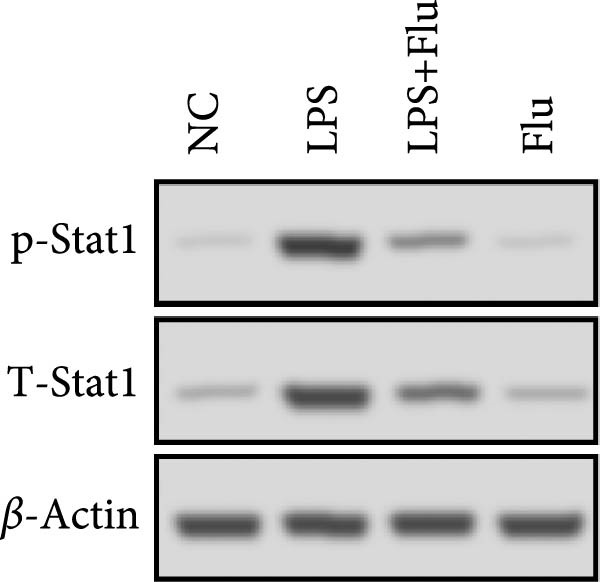
(A)

**Figure:**
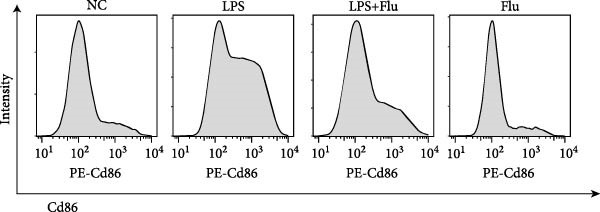
(B)

**Figure:**
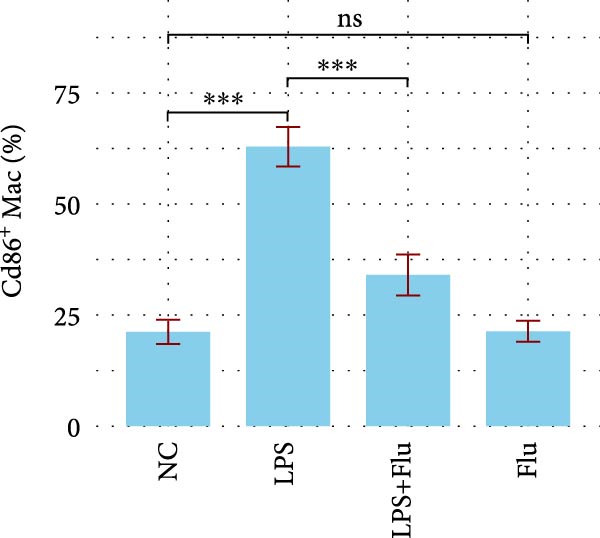
(C)

**Figure:**
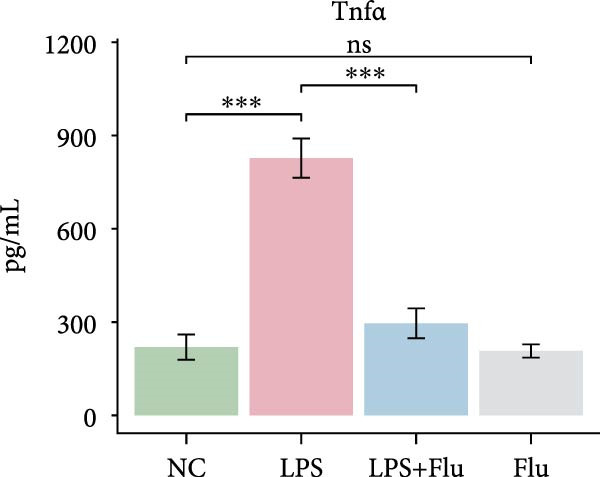
(D)

**Figure:**
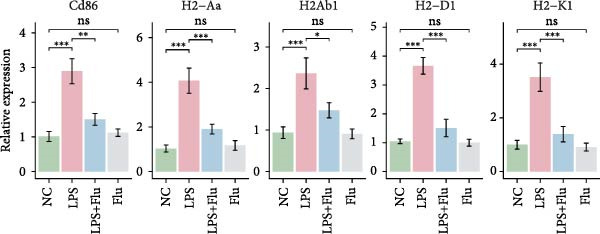
(E)

**Figure:**
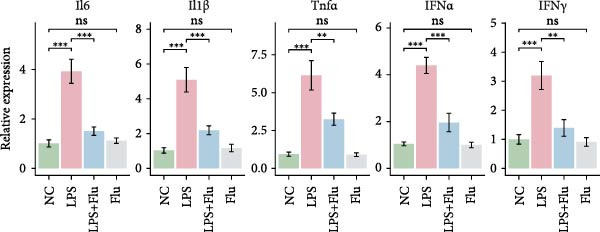
(F)

Flow cytometry revealed that LPS stimulation substantially increased macrophage activation, as indicated by elevated CD86 expression, whereas fludarabine‐mediated STAT1 inhibition significantly reduced these levels (Figure 6B,C), suggesting impaired T cell costimulatory capacity. Consistently, qRT‐PCR demonstrated that genes involved in antigen presentation and T cell costimulation—*H2-Aa*, *H2-Ab1*, *H2-D1*, *H2-K1*, and *Cd86*—were significantly downregulated following fludarabine treatment (Figure 6D). To evaluate inflammatory responses, ELISA results indicated that fludarabine significantly reversed LPS‐induced TNF‐*α* secretion in the culture medium (Figure 6E). Moreover, qRT‐PCR confirmed substantial reductions in LPS‐induced Tnf‐*α*, Il‐1*β*, Il‐6, Ifn‐*α*, and Ifn‐*γ* expression upon fludarabine treatment (Figure 6E).

Collectively, these findings demonstrate that STAT1 is a central regulator of macrophage‐mediated inflammation and antigen presentation. Pharmacological inhibition of STAT1 by fludarabine effectively attenuates both processes, underscoring its therapeutic potential in vitiligo.

## 4. Discussion

This study provides a comprehensive single‐cell transcriptomic characterization of macrophages in vitiligo and identifies a previously unrecognized Mac‐InflamAP subpopulation that plays a pivotal role in disease pathogenesis. Unlike prior work focusing predominantly on MELs and T lymphocytes in vitiligo, our data highlight macrophages as active orchestrators of both direct MEL injury and sustained immune activation. Specifically, Mac‐InflamAP cells exhibited terminal differentiation features, strong M1‐like polarization, and dual pathogenic functions—delivering TNF‐mediated cytotoxic signals to MELs and enhancing antigen presentation to T cells through elevated MHC‐I and MHC‐II expression. These findings expand the current immunopathological framework of vitiligo by positioning macrophages not merely as passive antigen‐presenting cells but as active disease‐promoting effectors.

The enrichment of Mac‐InflamAP in vitiligo lesions, coupled with their absence in healthy skin, suggests that local inflammatory cues drive macrophage reprogramming toward this pathogenic state. Pseudotime analysis indicated that Mac‐InflamAP emerges as a terminally differentiated subset, potentially derived from tissue‐resident or infiltrating macrophages under chronic inflammatory stress. This is consistent with recent observations in other autoimmune contexts, such as multiple sclerosis and rheumatoid arthritis, where late stage inflammatory macrophages display enhanced antigen‐presenting capacity and resistance to resolution signals [24, 25]. Our cell–cell communication analysis further supports this model, revealing Mac‐InflamAP as a central signaling hub in vitiligo skin, engaged in bidirectional activation loops with T cells and in TNF‐driven MEL suppression. While our single‐cell transcriptomic analysis comprehensively characterizes macrophage heterogeneity in vitiligo, it is important to note that the spatial localization of Mac‐InflamAP cells within lesional skin was not directly assessed in this study. Consequently, their precise proximity to MELs or T cells remains to be determined. Future studies employing spatial transcriptomics or multiplex immunofluorescence staining could provide critical insights into the spatial organization and cell–cell interactions of Mac‐InflamAP cells, particularly in active lesions. We sincerely acknowledge the reviewers’ thoughtful suggestions, which highlight the value of integrating spatial context to fully elucidate macrophage function in vitiligo pathogenesis.

Mechanistically, hdWGCNA identified STAT1 as a core transcriptional regulator linking inflammatory signaling and antigen presentation in Mac‐InflamAP. STAT1 has been implicated in macrophage activation and autoimmunity [26, 27], but its role in vitiligo had not been experimentally validated. A key strength of our work lies in the use of an unbiased, multistep bioinformatic pipeline—encompassing single‐cell subclustering, pseudotime inference, coexpression network analysis, and transcription factor prioritization—followed by in vivo and in vitro validation. This data‐driven approach minimizes prior assumptions and increases the robustness of identifying disease‐relevant regulators. Beyond vitiligo, such integrative analytic frameworks may serve as a methodological reference for dissecting cellular heterogeneity and uncovering therapeutic targets in other immune‐mediated disorders. Our in vivo and in vitro experiments show that pharmacological STAT1 inhibition by fludarabine curtails macrophage polarization, dampens antigen presentation, reduces T cell activation, and preserves MEL integrity. This complements earlier therapeutic strategies targeting JAK–STAT pathways in vitiligo [28, 29], but with a macrophage‐centered perspective. Importantly, the restoration of epidermal structure and melanogenesis in fludarabine‐treated mice underscores the potential of targeting macrophage‐intrinsic transcriptional programs to achieve durable repigmentation outcomes.

Clinically, these findings broaden therapeutic avenues beyond the conventional T cell‐directed or broadly anti‐inflammatory interventions. While systemic fludarabine carries toxicity concerns, our results raise the possibility of developing selective STAT1 inhibitors, topical formulations, or macrophage‐targeted delivery systems to modulate pathogenic macrophages while sparing systemic immunity. We acknowledge that fludarabine, as a systemic chemotherapeutic agent, has limited dermatologic applicability, and our experiments were not designed to evaluate dose–response relationships or alternative selective inhibitors. Future investigations could therefore explore safer and more targeted approaches to inhibit STAT1 activity. Moreover, the limited sample size and potential population bias in our current study may restrict the generalizability of the findings. Despite these limitations, our results provide a proof‐of‐concept that STAT1 represents a pivotal regulator of inflammatory macrophage activation in vitiligo. Given that current therapies such as JAK inhibitors often fail to achieve lasting repigmentation [29], macrophage‐targeted strategies may provide synergistic benefits when combined with T cell‐directed or MEL‐supportive treatments.

Nevertheless, this study has several limitations. First, the single‐cell data were derived from a relatively small cohort, which may not fully capture the heterogeneity of macrophage states across diverse patient populations or disease stages, potentially introducing population bias. Second, the spatial context of Mac‐InflamAP cells was not directly assessed, leaving open questions regarding their localization relative to T cells and MELs within lesional niches. Third, while in vivo experiments demonstrate that systemic fludarabine inhibits STAT1 and ameliorates disease, its known cytotoxicity and limited dermatologic applicability pose translational challenges. Additionally, lineage‐tracing and depletion models are required to clarify the ontogeny and plasticity of Mac‐InflamAP cells. Future studies integrating spatial transcriptomics, metabolic profiling, selective STAT1 inhibitors, and targeted delivery approaches will be essential to fully define the developmental trajectory, functional roles, and therapeutic vulnerabilities of this inflammatory macrophage subset.

## 5. Conclusion

In conclusion, we identify Mac‐InflamAP as a disease‐specific inflammatory macrophage subpopulation that integrates TNF‐mediated MEL inhibition with enhanced antigen presentation to T cells, thereby sustaining vitiligo‐associated autoimmunity. STAT1 emerges as a central driver of this phenotype, and its inhibition by fludarabine provides preclinical evidence for a macrophage‐targeted therapeutic strategy. These insights broaden the conceptual framework of vitiligo immunopathogenesis and open new avenues for intervention aimed at reprogramming pathogenic macrophage states.

## Ethics Statement

All experimental procedures were approved by the Institutional Animal Care and Use Committee of Beijing University of Chinese Medicine (Approval Number BUCM‐2024110103‐4077).

## Disclosure

All authors read and approved the final manuscript.

## Conflicts of Interest

The authors declare no conflicts of interest.

## Author Contributions

Ruozhou Qi and Min Huang designed the study, performed bioinformatics analyses, and drafted the manuscript. Ziyi Lin assisted with bioinformatics analyses. Huanhuan Deng and Yi Chen conducted the in vitro experiments, while Min Huang and Rule Sa performed the in vivo experiments. Xingwu Duan and Guangshan Chen supervised the project, provided critical revisions, and secured funding. Ruozhou Qi and Min Huang contributed equally to this work.

## Funding

This work was supported by the Clinical Research Fund of High‐Level Traditional Chinese Medicine Hospitals, Central Government (Project Number DZMG‐XZYY‐23009).

## Supporting Information

Additional supporting information can be found online in the Supporting Information section.

## Supporting information


**Supporting Information 1** Table S1: Primer sequences used for all qRT‐PCR experiments.

**Supporting Information 2** Table S2: Gene Ontology (GO) enrichment results for pseudotime regulatory genes.

**Supporting Information 3** Table S3: Genes comprising each hdWGCNA co‐expression module, along with their corresponding module membership scores.

**Supporting Information 4** This file contains three supporting figures. Figure S1: Expanded results for macrophage subset annotation and cross‐validation of macrophage subclusters using an independent scRNA‐seq dataset. Figure S2: Detailed results of cell–cell communication network analyses. Figure S3: Quality control plots and comprehensive module analysis for hdWGCNA.

## Data Availability

Single‐cell RNA‐seq data of healthy and vitiligo skins were obtained from Genome Sequence Archive (GSA) under Accession Number PRJCA006797 (https://ngdc.cncb.ac.cn/bioproject/browse/PRJCA006797).
