# Supplementary material for: Single‐Cell Transcriptomic Analysis Reveals an Inflammatory Antigen‐Presenting Macrophages Subtype Drive Vitiligo Pathogenesis Through STAT1‐Mediated Dual Mechanisms
Source: Mediators Inflamm. 2025 Dec 22;2025:8878698. doi: 10.1155/mi/8878698 (PMC12767439; doi:10.1155/mi/8878698)
Supplement: Supplementary file 4 — Supporting Information 4 This file contains three supporting figures. Figure S1: Expanded results for macrophage subset annotation and cross‐validation of macrophage subclusters using an independent scRNA‐seq dataset. Figure S2: Detailed results of cell–cell communication network analyses. Figure S3: Quality control plots and comprehensive module analysis for hdWGCNA. [file MI-2025-8878698-s002.pdf]

Figure S1. Macrophage subpopulation annotation and cross-validation using an independent scRNA-seq dataset.

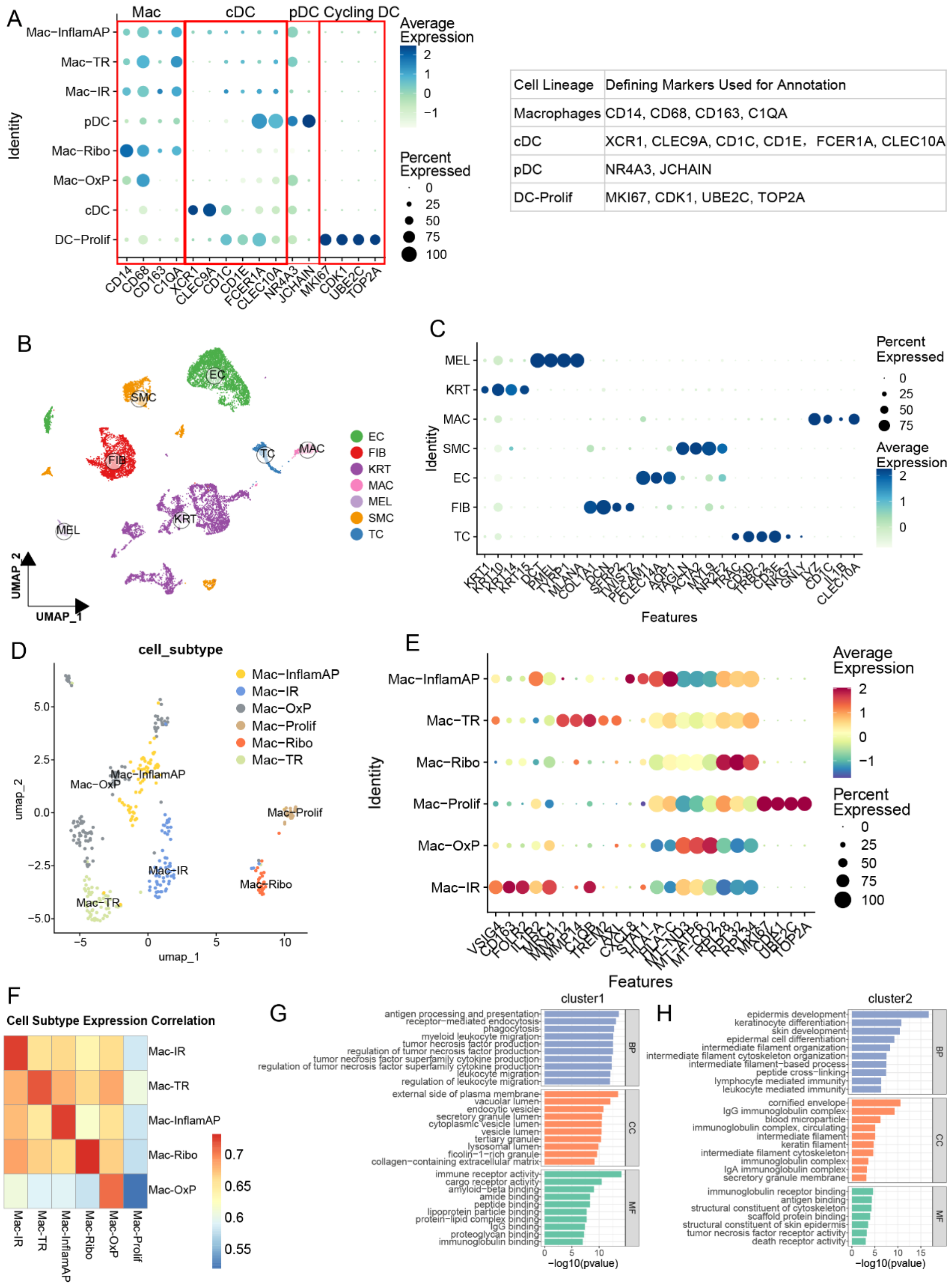

Cell Lineage	Defining Markers Used for Annotation
Macrophages	CD14, CD68, CD163, C1QA
cDC	XCR1, CLEC9A, CD1C, CD1E，FCER1A, CLEC10A
pDC	NR4A3, JCHAIN
DC-Prolif	MKI67, CDK1, UBE2C, TOP2A

(A) Dot plot showing the expression of marker genes across macrophage–DC subpopulations. Dot size represents the percentage of cells expressing each gene, and color intensity indicates the normalized expression level. The table on the right lists the marker genes for each subpopulation.

(B) UMAP visualization of an independent scRNA-seq dataset (GSE288871), with colors indicating

major cell types.

(C) Dot plot showing the expression of marker genes across the major cell types in the independent dataset. Dot size represents the percentage of cells expressing each gene, and color intensity indicates normalized expression.

(D) UMAP plot highlighting macrophage subpopulations in the independent scRNA-seq dataset (GSE288871), colored by subpopulation identity.

(E) Dot plot showing marker gene expression across macrophage subpopulations in the independent dataset. Dot size represents the percentage of cells expressing each gene, and color intensity indicates normalized expression.

(F) Heatmap of Pearson's correlation coefficients between macrophage subpopulations in the primary and validation datasets.

(G-H) Gene Ontology (GO) enrichment analysis for cluster 1 (G) and cluster 2 (H) genes. Bar plots show the top 10 significantly enriched GO terms, with colors indicating different GO categories and bar length representing the –log10(p-value) of enrichment.Figure S2. Cell–cell communication analysis reveals pathological signaling networks in vitiligo.

Figure S2. Cell–cell communication analysis reveals pathological signaling networks in vitiligo.

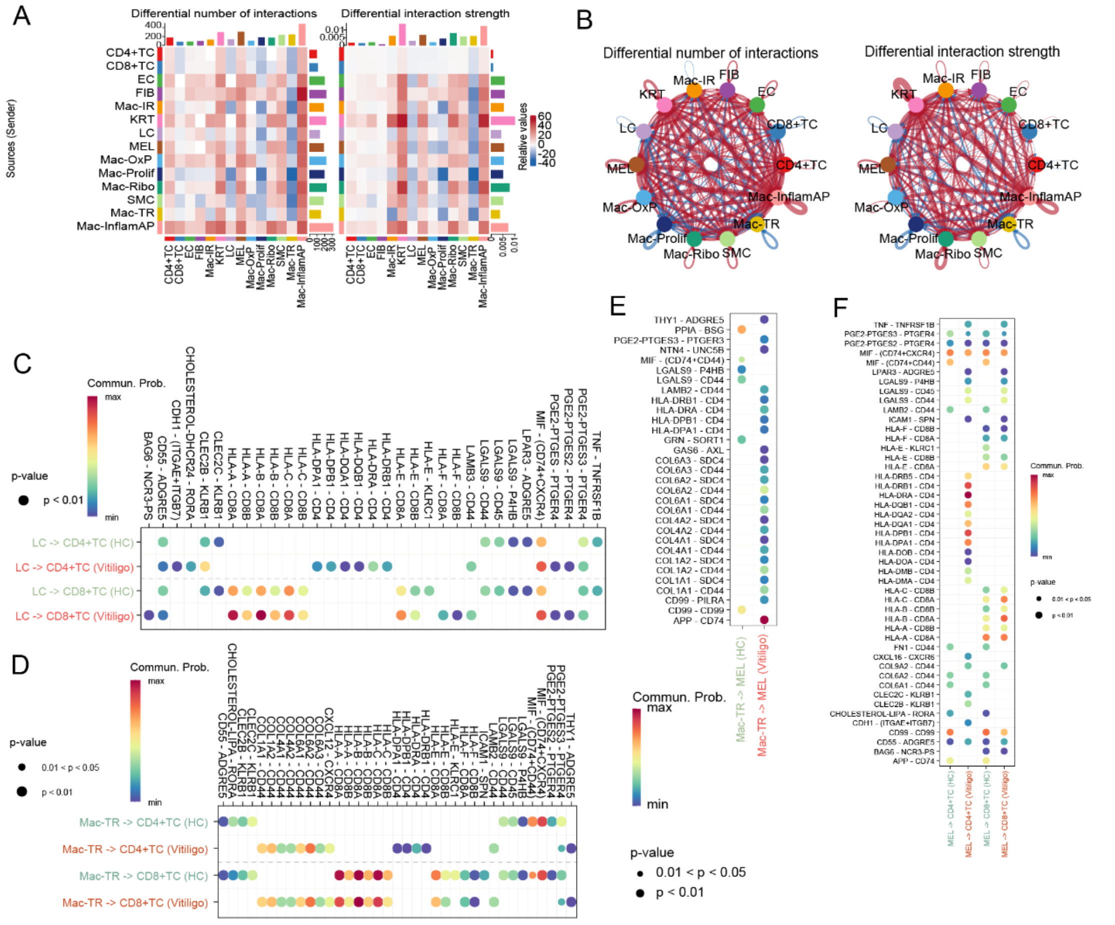

(**A**) Heatmaps showing differential numbers of interactions (left) and interaction strengths (right) between vitiligo and healthy controls.

(**B**) Circle plots depicting differential numbers of interactions (left) and interaction strengths (right) between vitiligo and healthy controls. Red edges indicate interactions enriched in vitiligo, while blue edges indicate those enriched in healthy controls.

(**C–F**) Dot plots illustrating communication probabilities from Langerhans cells (LCs) to T cells (**C**), tissue-remodeling macrophages (Mac-TRs) to T cells (**D**), Mac-TRs to melanocytes (MELs) (**E**), and MELs to T cells (**F**) in healthy controls and vitiligo samples. Dot color intensity represents the probability of interaction, and colored dots indicate pathways with significant enrichment ($p < 0.05$).

Figure S3. hdWGCNA reveals macrophage-specific regulatory modules and hub genes associated with potential therapeutic targets.

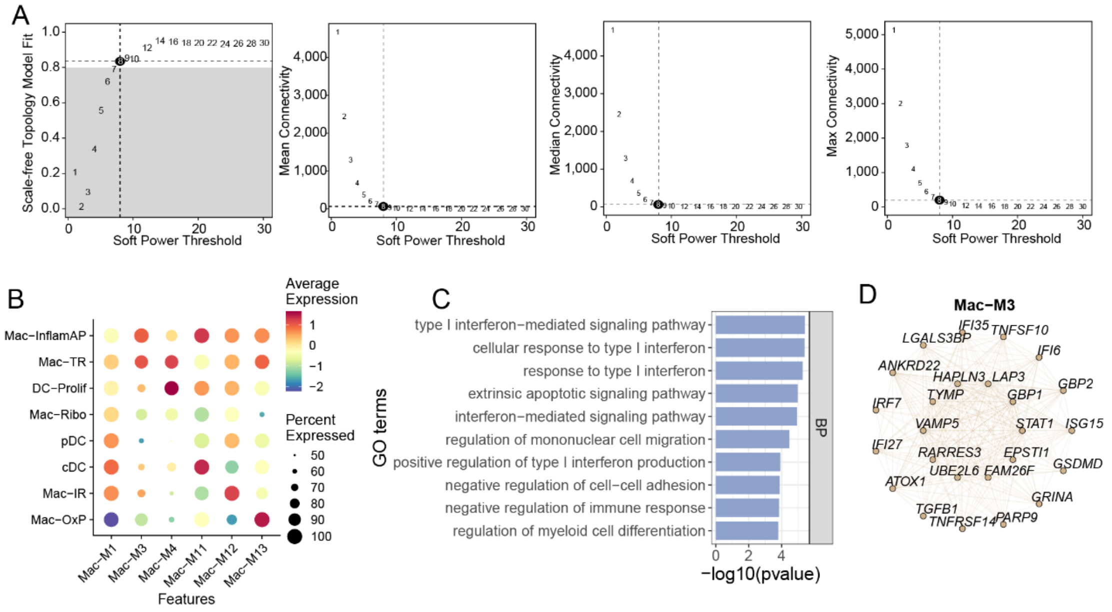

(**A**) Soft-thresholding power selection for hdWGCNA. The left panel shows scale-free topology model fit (R^2) across different powers, with the grey zone indicating the threshold ($R^2 = 0.8$). The right panels display connectivity metrics, with $\beta = 8$ chosen as the optimal power.

(**B**) Dot plot showing the expression patterns of macrophage-enriched modules across macrophage subpopulations. Dot color intensity represents the average expression level.

(**C**) Bar plot of top enriched Gene Ontology (GO) biological processes for genes in the Mac-M3 module.

(**D**) Network visualization of hub genes within the Mac-M3 module.
